# Lactylation Enhances the Activity of Lactate Dehydrogenase A and Promotes the Chemoresistance to Cisplatin Through Facilitating DNA Nonhomologous End Junction in Lung Adenocarcinoma

**DOI:** 10.1002/advs.202510733

**Published:** 2025-11-05

**Authors:** Jizhuo Li, Wenze Xun, Xijie Wang, Ruiguang Luo, Qifan Hu, Zhaocai Zhou, Jian Yuan, Yanan Wang, Xiaorui Wan, Tao Zhao, Tianyu Han, Jian‐Bin Wang

**Affiliations:** ^1^ Department of Thoracic Surgery The First Affiliated Hospital of Nanchang University Nanchang Jiangxi 330006 China; ^2^ The MOE Basic Research and Innovation Center for the Targeted Therapeutics of Solid Tumors Jiangxi Medical College Nanchang University Nanchang 330031 China; ^3^ Jiangxi Provincial Key Laboratory of Tumor Biology Nanchang University Nanchang Jiangxi 330031 China; ^4^ School of Basic Medical Sciences Nanchang University Nanchang Jiangxi 330031 China; ^5^ State Key Laboratory of Genetics and Development of Complex Phenotypes School of Life Sciences Zhongshan Hospital Fudan University Shanghai 200438 China; ^6^ State Key Laboratory of Cardiology and Research Center for Translational Medicine Shanghai East Hospital Tongji University School of Medicine Shanghai 200120 China; ^7^ Jiangxi Provincial Key Laboratory of Respirtory Diseases Jiangxi Institute of Respiratory Disease The Department of Respiratory and Critical Care Medicine The First Affiliated Hospital Jiangxi Medical College Nanchang University Nanchang Jiangxi 330006 China; ^8^ Jiangxi Clinical Research Center for Respiratory Diseases Nanchang Jiangxi 330006 China; ^9^ China‐Japan Friendship Jiangxi Hospital National Regional Center for Respiratory Medicine Nanchang Jiangxi 330006 China

**Keywords:** DNA damage repair, glycolysis, lactylation, LDHA

## Abstract

Lactylation is a novel post‐translational modification closely related to the glycolytic process, but the regulatory mechanisms between lactylation and glycolysis are far from being elucidated. Lactate dehydrogenase A (LDHA) catalyzes the formation of lactate, which provides the modifying group for protein lactylation. However, whether lactylation occurs on LDHA itself remains unknown. Here, it is found that lactylation promotes the enzymatic activity of LDHA in lung adenocarcinoma (LUAD), which in turn enhances the overall level of cellular lactylation through a positive feedback loop. Screening identified Lys81 and Lys318 as key LDHA lactylation sites, with alanyl‐tRNA synthetase 1 (AARS1) serving as the mediating lactyltransferase. Mass spectrometry reveals that numerous proteins involved in DNA nonhomologous end junction (NHEJ), including FEN1, XRCC5, and XRCC6 might be regulated by lactylation. Delactylation of these proteins significantly hinders the formation of FEN1‐RAD1‐RAD9A‐HUS1 complex, thereby leading to dysfunction of NHEJ and increasing the sensitivity of cancer cells to cisplatin. In summary, this work identifies LDHA lactylation as a critical mechanism for accelerating the progression of LUAD and reveals how this lactylation influenced cisplatin sensitivity of LUAD cells, which deepen the understanding of lactylation‐mediated tumor progression and provide a potential new anticancer strategy.

## Graphical abstract

Lactylation, linked to glycolysis, is poorly understood in regulation. It is found that LDHA is lactylated at Lys81/Lys318 by AARS1, boosting its activity and cellular lactylation via positive feedback in LUAD. Lactylation regulates NHEJ‐related proteins; their delactylation impairs NHEJ and increases cisplatin sensitivity, offering anticancer insights.

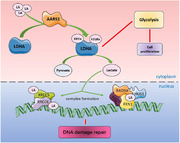

## 1 Introduction

Metabolic reprogramming stands as a hallmark of cancer.^[^ ^1^, ^2^ ^]^ Unlike normal cells, tumor cells tend to rapidly generate energy through glycolysis even in aerobic environments, a phenomenon known as the Warburg effect, which is one of the key factors that enable tumor cells to maintain high proliferation rates.^[^ ^3^, ^4^ ^]^ Lactate, as a metabolic byproduct of glycolysis, functioned not only as an energy substrate and signaling molecule,^[^ ^5^ ^]^ but also a modifying group for the newly discovered post‐translational modification‐lactylation. Lactylation can occur on both histones and nonhistone proteins. There is substantial evidence indicating that lactylation plays a significant role in neural excitation,^[^ ^6^ ^]^ macrophage polarization,^[^ ^7^ ^]^ innate immune defence,^[^ ^8^ ^]^ and tumor development.^[^ ^9^ ^]^ In tumors, lactylation has been demonstrated to influence tumor progression by regulating resistance to chemotherapy,^[^ ^10^ ^]^ malignancy alterations,^[^ ^11^ ^]^ autophagy,^[^ ^12^ ^]^ and anti‐tumor immunity.^[^ ^13^ ^]^

Lactate dehydrogenase (LDH) is a tetramer composed of two distinct subunits, LDHA and LDHB. It is widely believed that LDHA has a high affinity for pyruvate, primarily catalyzing the reduction of pyruvate to lactate, while LDHB has a higher affinity for lactate, primarily catalyzing the oxidation of lactate to pyruvate.^[^ ^14^ ^]^ As a pivotal regulator of the Warburg effect, LDHA has emerged as a widely investigated therapeutic target for tumors. Post‐translational modification (PTM) plays an important role in regulating LDHA protein function. For example, sirtuin 2 (SIRT2) promoted the enzymatic activity of LDHA by decreasing its acetylation.^[^ ^15^ ^]^ Palmitoylation altered LDHA activity and the response to chemotherapy of pancreatic cancer.^[^ ^16^ ^]^ Succinylation attenuated the proteasomal degradation of LDHA by affecting its protein stability.^[^ ^17^ ^]^ Thus, clarifying the regulatory mechanisms of PTM on LDHA in cancer is important for us to deeply understand the role of LDH in tumor progression.

Genomic instability is another hallmark of cancers. The rapid proliferation of tumor cells is accompanied by an increased accumulation of DNA damage. To counteract this, DNA damage repair is particularly active in tumor cells, and the sensitivity of cancer cells to radiotherapy or chemotherapy is also based on this characteristic.^[^ ^18^, ^19^ ^]^ Some evidence have shown that protein lactylation might be an important regulatory factor in DNA damage repair. For instance, lactylation of Nijmegen breakage syndrome protein1 (NBS1) at lysine 388 (K388) was essential for MRE11–RAD50–NBS1 (MRN) complex formation and the accumulation of homologous recombination (HR) repair proteins at the sites of DNA double‐strand breaks.^[^ ^10^ ^]^ According to another study, meiotic recombination 11 homolog 1 (MRE11) lactylation promoted its binding to DNA, facilitating DNA end resection and HR. A cell‐penetrating peptide that specifically blocked MRE11 lactylation inhibited HR and sensitized cancer cells to cisplatin and poly ADP‐ribose polymerse inhibitor.^[^ ^20^ ^]^ However, it remains unknown whether lactylation could affect nonhomologous end joining (NHEJ).

Herein, we demonstrate that lactylation of LDHA is an important carcinogenic factor in LUAD. Mechanistically, LDHA can be lactylated leading to the enhanced enzymatic activity. We elucidated that K81 and K318 of LDHA were the key lactylation sites and that AARS1 was the lactyltransferase responsible for this lactylation. We also found that inhibiting the lactylation of LDHA decreased the global protein lactylation, including proteins involved in NHEJ, and sensitized LUAD cells to cisplatin by affecting the formation of the FEN1‐RAD1‐RAD9A‐HUS1 complex. Thus, this study elucidates the regulatory mechanism between lactylation and glycolysis, clarifying the molecular mechanism through which LDHA regulates NHEJ through lactylation in LUAD, thereby providing a theoretical basis for developing antitumor drugs by targeting LDHA.

## 2 Results

### 2.1 LDHA, Rather Than LDHB, is the Key Factor Driving Global Lactylation in LUAD Cells

**Figure 1:**
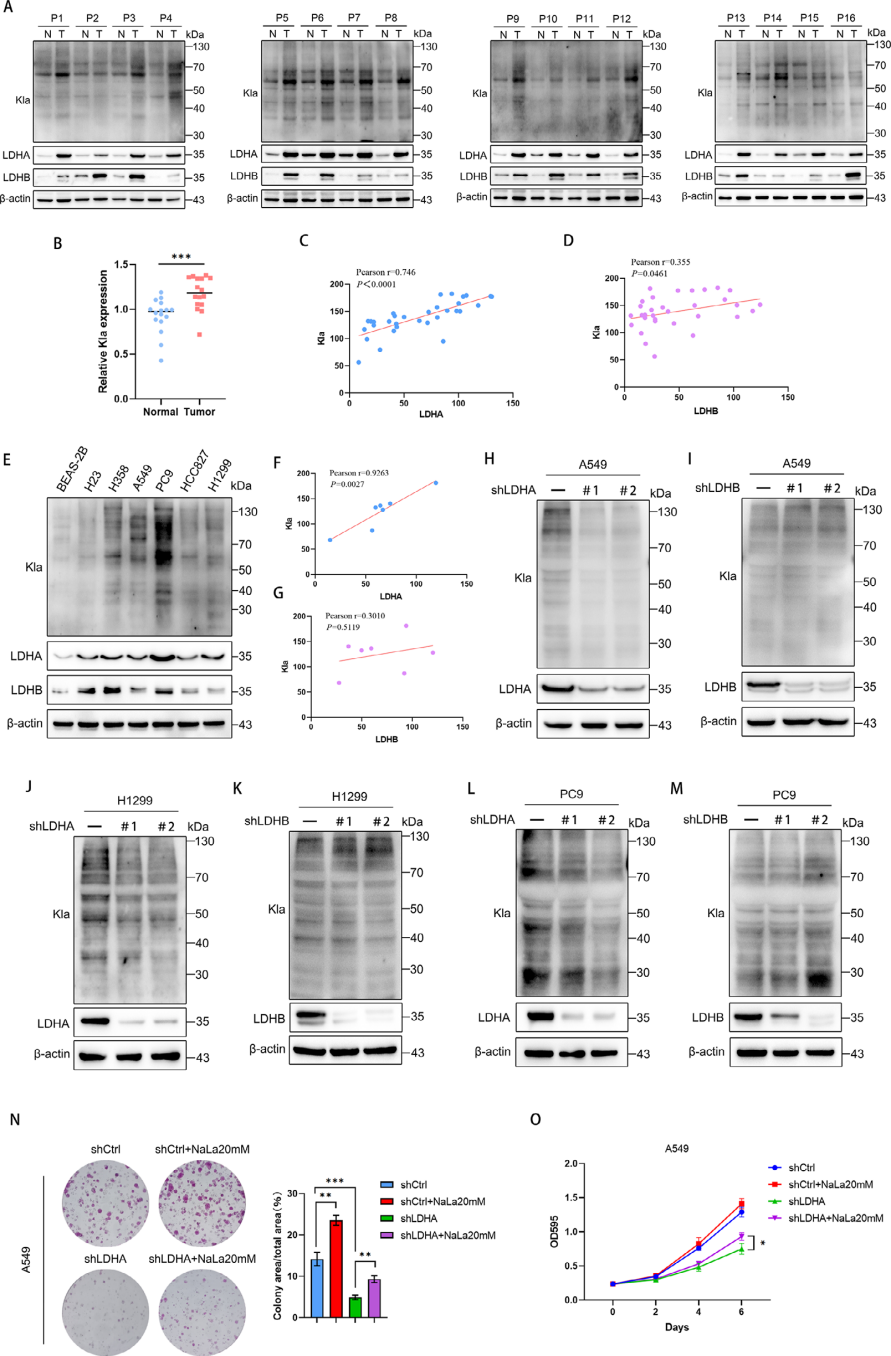
LDHA, rather than LDHB, is the key factor driving global lactylation in LUAD cells. A) Protein levels of Kla, LDHA, and LDHB were measured by Western blotting in paired LUAD tissues (T) and adjacent normal tissues (N). B) Kla expression differences between LUAD (T) and normal tissues (N) were assessed by Student's *t*‐test. Pearson correlation analysis was conducted for Kla‐LDHA, C) and Kla‐LDHB D) expression. E) Western blot analysis of differential expression of Kla, LDHA, and LDHB between human bronchial epithelial cell line BEAS‐2B and LUAD cell lines. F) Pearson correlation analysis was performed to assess the relationships between Kla expression and LDHA (F)/LDHB (G) levels in the cell lines. H) Protein lactylation levels were compared between control and LDHA‐knockdown (shLDHA) or LDHB‐knockdown (shLDHB). I) A549 cells by Western blotting using anti‐lactyllysine antibody. J) Protein lactylation levels were compared between control and LDHA‐knockdown (shLDHA) or LDHB‐knockdown (shLDHB). K) H1299 cells by Western blotting using anti‐lactyllysine antibody. L) Protein lactylation levels were compared between control and LDHA‐knockdown (shLDHA) or LDHB‐knockdown (shLDHB). M) PC9 cells by Western blotting using anti‐lactyllysine antibody. N) Colony formation assay of A549‐shCtrl and A549‐shLDHA cells treated with 0 or 20 mm NaLa. O) Growth curve analysis of A549‐shCtrl and A549‐shLDHA cells treated with 0  or 20 mm NaLa. Data are presented as mean ± SD from three independent experiments. ^*^ *p* < 0.05, ^**^ *p* < 0.01; ns stands for no significant change.

To investigate whether lactylation is enhanced in LUAD, we detected the lactylation in the tissues of 16 LUAD patients, Western blot analysis revealed that LUAD tissues exhibited significantly higher pan‐lysine lactylation (Kla) than normal tissues (**Figure** **1**A,B). LDHA prefers to catalyze the reduction of pyruvate to lactate, whereas LDHB has higher activity in the oxidation of lactate to pyruvate.^[^ ^14^, ^21^ ^]^ Therefore, we speculate that LDHA is the key molecule driving lactylation. To validate this hypothesis, we examined the expression of LDHA and LDHB in LUAD tissues and analyzed their correlation with Kla. The results indicated that the expression of LDHA and LDHB in LUAD tissues were higher than those in adjacent normal tissues, but LDHA showed a stronger correlation with Kla (Figure 1C,D). In addition, similar results were obtained when we examined the expression of Kla and LDHA in the human bronchial epithelial cell line BEAS‐2B and LUAD cells (Figure 1E–G).

To further detect the effects of LDHA in the regulation of cell global lysine lactylation, we knocked down LDHA and LDHB in LUAD cells (A549, H1299 and PC9) and found that knockdown of LDHA inhibited global lactylation, whereas knockdown of LDHB had no similar effect (Figure 1H–M). Furthermore, knockdown of LDHA exhibited more pronounced inhibitory effects on the proliferation and migration of LUAD cells than LDHB knockdown (Figure S1A–E, Supporting Information). As a key enzyme downstream of glycolysis, LDHA has been shown to promote multiple types of tumor progression,^[^ ^22^, ^23^, ^24^, ^25^, ^26^ ^]^ to explore whether LDHA promoted LUAD progression by regulating global lysine lactylation, LDHA‐knockdown A549 and H1299 cells were cultured with 20 mm lactate sodium (NaLa) and found that LDHA silencing exhibited significant inhibition of cell proliferation and migration, while NaLa attenuated the inhibitory effect of LDHA silencing (Figure 1N,O; Figure S1F–K, Supporting Information). Together, these findings indicate that LDHA, not LDHB, is the key regulator of global lysine lactylation and promotes the progression of LUAD through lactylation.

### 2.2 The Lactylation of LDHA is Crucial for Maintaining Its Enzymatic Activity

**Figure 2:**
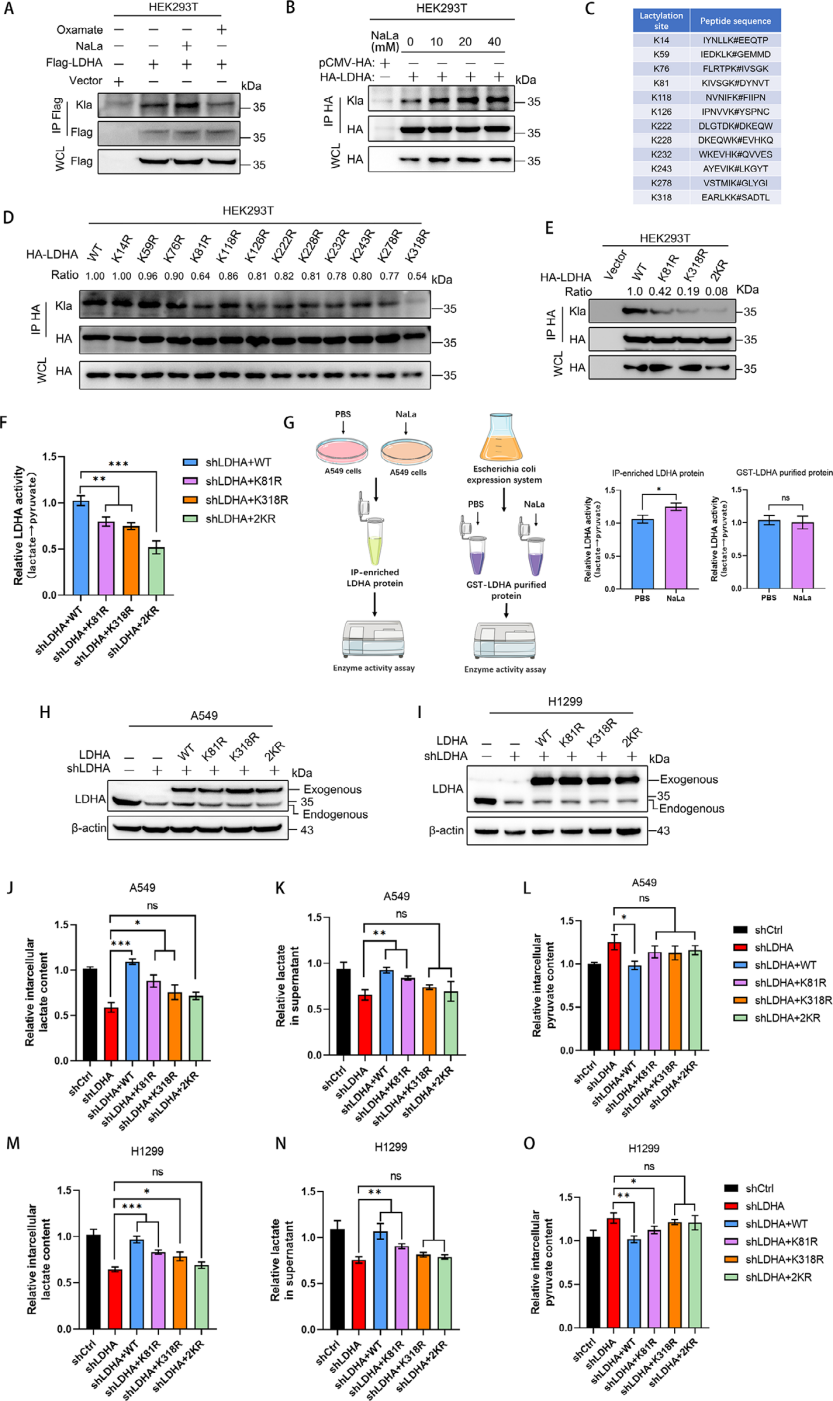
The lactylation of LDHA is crucial for maintaining its enzymatic activity. A) Measurement of LDHA lactylation in HEK293T cells. Cells transfected with LDHA were treated with 20 mm NaLa or 20 mm Oxamate for 24 h. Immunoprecipitation was performed and lactylation was analyzed by Western blot with Pan‐Kla antibody. B) HEK293T cells transfected with LDHA were treated with increasing concentrations of NaLa (0, 10, 20, or 40 mm) for 24 h. LDHA was immunoprecipitated and lactylation levels were detected by Western blotting using pan‐Kla antibody. C) LDHA lactylation sites through integrated analysis of published lactylome datasets. D) Lactylation analysis of wild‐type LDHA and its mutants in HEK293T cells. E) Lactylation assay in HEK293T cells (LDHA‐WT, LDHA‐K81R, LDHA‐K318R and LDHA‐2KR) was analyzed by Western blot. F) In vitro LDHA activity assay. Flag‐tagged LDHA variants (WT, K81R, K318R, and 2KR) were purified from HEK293T cells using anti‐Flag beads. Enzymatic activity was measured by monitoring NADH oxidation at 340 nm in a microplate assay with pyruvate as substrate. G) Detection of LDHA activity following either IP of LDHA protein from A549 cells treated with PBA or NaLa, or after in vitro co‐incubation of prokaryotically expressed and purified LDHA protein with PBA or NaLa. H,I) In A549 (H) and H1299 cells (I), the endogenous LDHA was replaced by shRNA‐resistant and FLAG‐tagged LDHA WT or mutants (K81R, K318R, 2KR). The expressions of LDHA were examined by Western blot. J) Measurement of intracellular and extracellular lactate levels K) of A549 cell variants in H. using a lactate assay kit. L) Measurement of intracellular pyruvate levels of A549 cell variants in H. using a pyruvate assay kit. M,N) Measurement of lactate levels in cytoplasm and culture medium (N) of H1299 cell variants using a lactate assay kit. O) Measurement of intracellular pyruvate levels in H1299 cell variants. Data are presented as mean ± SD from three independent experiments. ^*^ *p* < 0.05, ^**^ *p* < 0.01, ^***^ *p* < 0.001; ns stands for no significant change.

LDHA regulates the overall lactylation of cells, but there have been no reports on whether lactylation regulates the function of LDHA protein. As LDHA directly catalyzes the production of lactate, we speculated that LDHA might also undergo lactylation and play an important role in regulating its protein function. To verify this hypothesis, LDHA‐Flag was purified after immunoprecipitation from human embryonic kidney HEK293T cells treated with 20 mm NaLa or 20 mm Oxamate for 24 h and analyzed by Western blot, the results showed that the lactylation of LDHA could be detected using the pan‐Kla antibody, and NaLa enhanced this lactylation of LDHA, while the LDHA inhibitor‐Oxamate significantly inhibited the lactylation of LDHA (**Figure** **2**A). Moreover, with the increase in the concentration of NaLa, the lactylation level of LDHA also gradually increased (Figure 2B), indicating that the lactylation of LDHA was directly regulated by the lactate content in LUAD cells.

To further investigate the key lactylation sites of LDHA, we compiled the global lysine lactylome from recent publications and identified 12 potential lactylation sites in LDHA (Figure 2C).^[^ ^27^, ^28^, ^29^ ^]^ Subsequently, we mutated all 12 lactylation sites and transfected them into HEK293T cells to assess the differences in lactylation levels between the mutants and the wild‐type (WT). The results revealed that only the mutation of lysine 81 (K81) and lysine 318 (K318) to arginine led to the most significant downregulation of LDHA lactylation (Figure 2D), and simultaneous mutation of K81 and K318 nearly completely abolished the lactylation of LDHA (Figure 2E). Additionally, K81 and K318 are highly conserved across multiple species (Figure S2A, Supporting Information). Acetylation also occurs on lysine residues and might interfere with lactylation at the same site, so we investigated whether K81 and K318 were critical for LDHA acetylation. Surprisingly, the mutations at K81 and K318 did not alter the acetylation levels of LDHA (Figure S2B, Supporting Information), indicating that K81 and K318 were not key acetylation sites of LDHA. To investigate whether lactylation was involved in regulating the protein function of LDHA, we first assessed the protein degradation rates. LDHA‐WT, LDHA‐K81R, and LDHA‐K318R were transfected into A549 cells, respectively. Subsequently, upon treatment with cycloheximide (CHX), no significant differences in degradation were observed among LDHA‐WT, LDHA‐K81R, and LDHA‐K318R (Figure S2C–E, Supporting Information). In addition, after treating A549 and H1299 cells with varying concentrations of sodium lactate (NaLa), no significant changes in the protein levels of LDHA were observed (Figure S2F,G, Supporting Information), indicated that lactylation does not regulate the expression or degradation of LDHA. Next, we sought to examine whether K81 and K318 lactylation modulates the enzymatic activities of LDHA, Flag‐tagged LDHA‐WT, LDHA‐K81R, LDHA‐K318R, and LDHA‐2KR were individually transfected into HEK293T cells. Proteins were immunoprecipitated using an anti‐Flag antibody, and LDHA enzymatic activity was assessed in vitro. The results revealed that, compared to LDHA‐WT, the enzymatic activities of LDHA‐K81R and LDHA‐K318R decreased, whereas the activity of LDHA‐2KR showed a more significant reduction (Figure 2F). To exclude the potential influence of lactate acting as a product to inhibit LDHA activity, A549 cells were treated with PBS or NaLa, respectively. It was observed that NaLa treatment significantly enhanced intracellular LDHA activity. However, direct incubation of prokaryotically expressed and purified LDHA protein with NaLa did not alter its enzymatic activity (Figure 2G), indicating that the increase in LDHA activity induced by NaLa is dependent on lactoyltransferases present in cells. These findings suggest that lactylation modification exerts a far greater impact on LDHA enzymatic activity than does lactate acting merely as a product of the enzymatic reaction. Furthermore, LDHA lactylation mutant cell lines were constructed which the endogenous LDHA was first depleted using shRNA and subsequently replaced with shRNA‐resistant and FLAG‐tagged LDHA‐WT, LDHA‐K81R, LDHA‐K318R, or LDHA‐2KR mutants (Figure 2H,I). Then the intracellular and extracellular lactate levels, as well as the intracellular pyruvate levels, were measured. Surprisingly, knockdown of LDHA significantly reduced both intracellular and extracellular lactate levels and led to a marked accumulation of intracellular pyruvate. Overexpression of exogenous LDHA‐WT was able to rescue the effects caused by the knockdown, whereas LDHA‐2KR failed to exert the same effect, indicating that lactylation is crucial for maintaining the enzymatic activity of LDHA (Figure 2J–O). To investigate the clinical significance of LDHA lactylation, we detected lactylation of LDHA in paired tumor and adjacent normal tissues from four patients with lung adenocarcinoma using immunoprecipitation. The results demonstrated a higher level of LDHA lactylation in tumor tissues compared to adjacent normal tissues (Figure S2H, Supporting Information).

### 2.3 The Loss of Lactylation in LDHA Impairs Its Carcinogenic Ability

**Figure 3:**
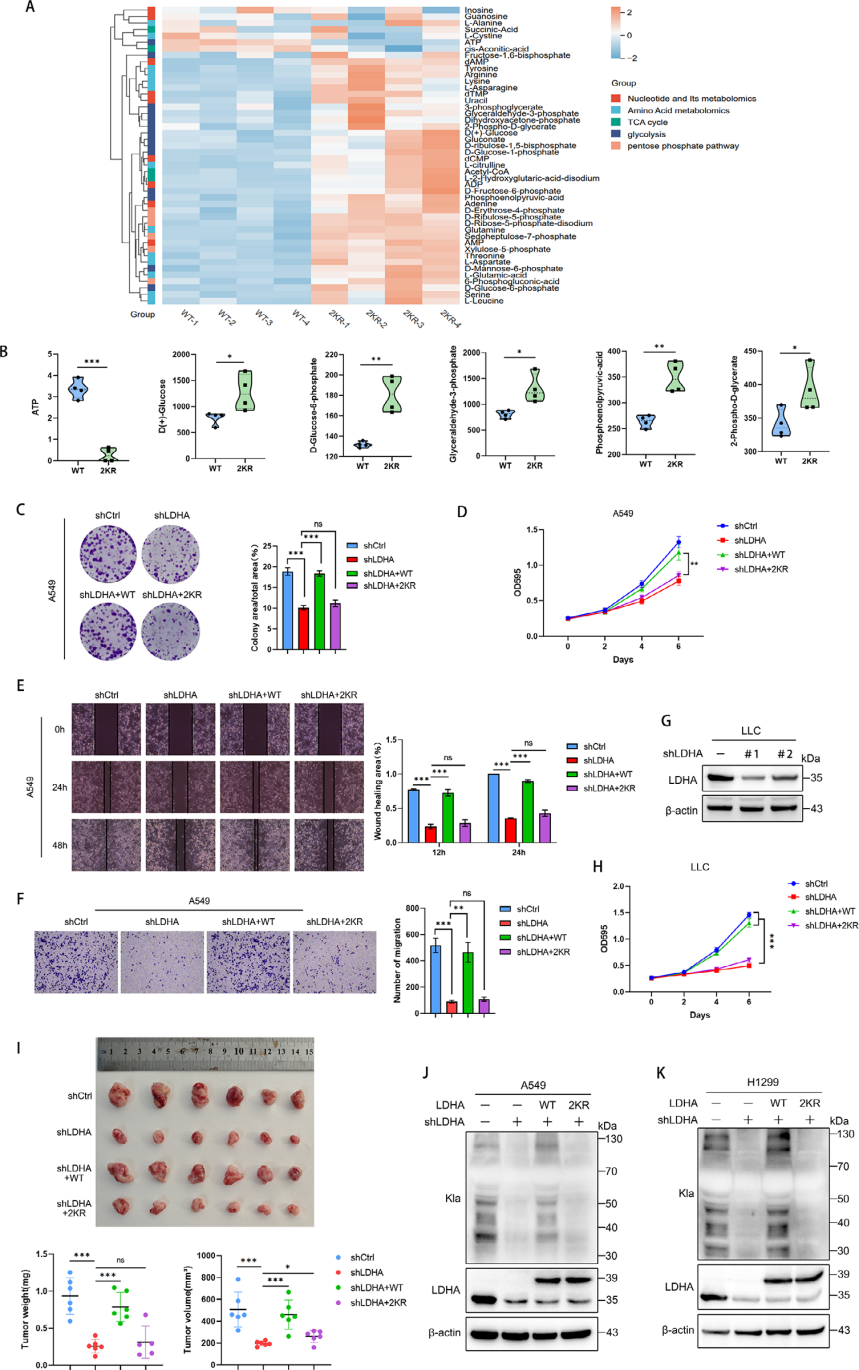
The loss of lactylation in LDHA impairs its carcinogenic ability. A) Reintroduction of exogenous LDHA‐WT and LDHA‐2KR into endogenous LDHA‐knockdown A549 cells for targeted energy metabolomics analysis. B) Major differentially altered metabolites in the glycolytic pathway. C) A549 cells were transduced with LDHA‐targeting shRNA or control shRNA, followed by expression of shRNA‐resistant LDHA constructs (WT or 2KR). Colony formation was assessed by crystal violet staining after 10 days. D) Cell proliferation was measured by growth assay in different A549 cells. E) Cell horizontal migration was assessed by scratch wound healing assay in various A549 cell lines. Wound closure was monitored at 0, 24, and 48 h post‐scratching. F) Transwell migration assay to evaluate vertical migration capacity of different A549 cell variants. G) In LLC cells, LDHA was knocked down by shRNA. H) Reintroduction of exogenous LDHA‐WT and LDHA‐2KR into LDHA knockdown cell (shLDHA#1) of G, the differences of cells proliferation were detected. I) LLC‐shCtrl, LLC‐shLDHA, LLC‐shLDHA+WT or LLC‐shLDHA+2KR cells (4 × 10^5^) were subcutaneously injected into 5‐week‐old C57BL/6 mice. After 16 days, the tumors were dissected and photographed. The weight and volume of allograft tumors were measured. J,K) Western blot analysis of lactylation levels in different A549 and H1299 cell variants. Data are presented as mean ± SD from three independent experiments. ^*^ *p* < 0.05, ^**^ *p* < 0.01, ^***^ *p* < 0.001; ns stands for no significant change.

LDHA is a key enzyme in the glycolytic pathway and a critical driver of the Warburg effect in tumor cells. Thus, we hypothesize that the lactylation level of LDHA might influence tumor proliferation by modulating glycolysis. Following endogenous LDHA knockdown by shRNA in A549 cells, we reconstituted the A549 cells with shRNA‐resistant LDHA‐WT or LDHA‐2KR constructs. Targeted energy metabolomics was then performed to analyze metabolic differences between these two cell lines (**Figure** **3**A; Table S1, Supporting Information). The results revealed that, compared to A549‐LDHA‐WT cells, A549‐LDHA‐2KR cells exhibited an accumulation of upstream glycolytic metabolites (D (+) Glucose, D‐Glucose‐6‐phosphate, Glyceraldehyde‐3‐phosphate, Phosphoenolpyruvic‐acid and 2‐Phospho‐D‐glycerate) and a significant reduction in ATP production (Figure 3B), indicating that the loss of LDHA lactylation severely impaired glycolysis in LUAD.

Subsequently, we investigated the impact of LDHA lactylation deficiency on its oncogenic functions. Both cell proliferation assays and colony formation assays demonstrated that knockdown of LDHA significantly inhibited the proliferative capacity of A549 and H1299 cells. Overexpression of exogenous LDHA‐WT was able to rescue the proliferative suppression caused by the knockdown, whereas exogenous LDHA‐2KR failed to restore this effect (Figure 3C,D; Figure S3A,B, Supporting Information). Similarly, wound healing assays and Transwell assays used to assess cell migration capacity yielded consistent results. Only the overexpression of exogenous LDHA‐WT, but not LDHA‐2KR, was able to rescue the migration suppression caused by LDHA knockdown in A549 and H1299 cells (Figure 3E,F; Figure S3C,D, Supporting Information). To evaluate the oncogenic role of LDHA lactylation in vivo, we generated mice Lewis lung carcinoma (LLC) cells with stable LDHA knockdown and complemented them with exogenous overexpression of LDHA‐WT or LDHA‐2KR (Figure 3G; Figure S3E, Supporting Information). These modified LLC cells were then subcutaneously injected into C57BL/6 mice. Consistent with the in vitro observations, the reduced tumor development in LDHA‐depleted cells was rescued only by overexpression of LDHA‐WT, whereas LDHA‐2KR failed to restore tumor growth (Figure 3H,I; Figure S3F, Supporting Information). The results revealed that deficiency in LDHA lactylation also significantly suppressed tumor growth in vivo. Moreover, immunohistochemical staining revealed stronger Ki67 and pan‐lactylation (Kla) signals in tumor tissues derived from shCtrl and shLDHA+WT LLC cells compared to those from shLDHA and shLDHA+2KR LLC cells (Figure S3G, Supporting Information).

The activity of LDHA directly influences the overall cellular lactate levels, and our study demonstrated that lactylation regulated LDHA activity. Therefore, it is hypothesized that there existed a positive feedback regulatory mechanism between lactylation and LDHA. To further validate this, the global protein lactylation levels were measured in A549, H1299 and LLC cells transfected with shCtrl, shLDHA, shLDHA+WT, or shLDHA+2KR using the Pan‐Kla antibody. The results revealed that, compared to LDHA‐WT, LDHA‐2KR failed to rescue the downregulation of global protein lactylation caused by LDHA knockdown (Figure 3J,K; Figure S3E, Supporting Information). These results suggest that lactylation promotes the carcinogenicity of LDHA through positive feedback regulation.

### 2.4 AARS1 is the Lactyltransferase of LDHA and Enhances Its Enzymatic Activity

To date, multiple lactyltransferases have been identified, among which E1A binding protein p300 (p300) was the first discovered lactyltransferase capable of broadly mediating lactylation modifications on both histone and nonhistone proteins in cells.^[^ ^7^, ^30^ ^]^ To investigate whether p300 was also involved in regulating the lactylation of LDHA, we co‐overexpressed p300 and HA‐LDHA in A549 cells. Following immunoprecipitation, HA‐LDHA was purified from the cells, and the changes in lactylation modifications were assessed. The results demonstrated that overexpression of p300 significantly upregulated global lactylation levels in A549 cells but had no effect on the lactylation of LDHA (Supplementary Figure S4A,B), indicating that p300 was not the lactyltransferase responsible for LDHA lactylation.

**Figure 4:**
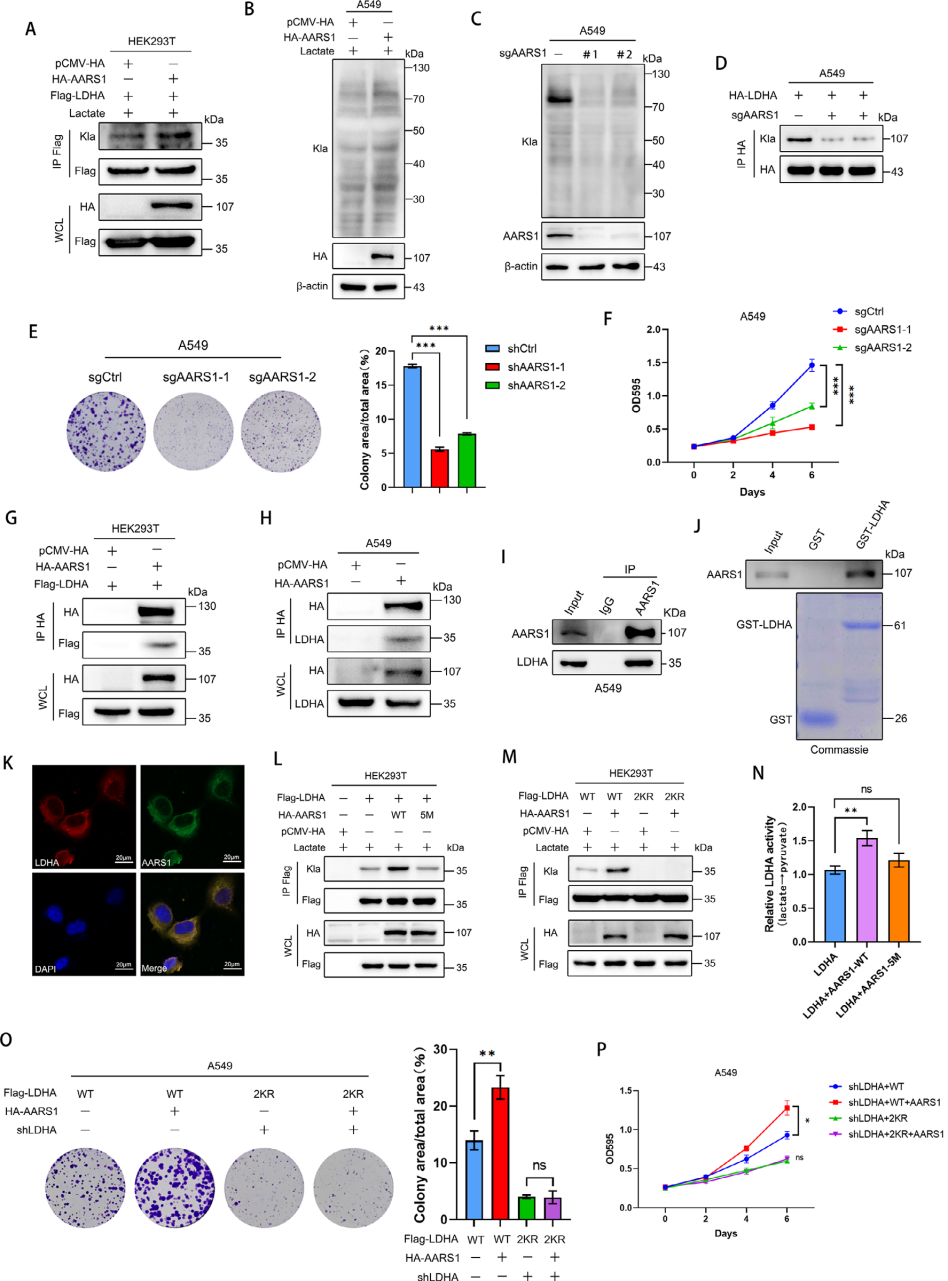
AARS1 is the lactyltransferase of LDHA and enhances its enzymatic activity. A) Co‐IP analysis of LDHA lactylation in HEK293T cells overexpressing AARS1 under lactate treatment. B) Global lactylation profiling in AARS1‐overexpressing A549 cells. C) Global lactylation analysis in AARS1‐knockout A549 cells. D) Co‐IP analysis of LDHA lactylation in A549 cells after control knockout or AARS1 knockout. E) Colony formation ability of AARS1‐knockout A549 cells. F) Cell proliferation analysis of A549 cells following AARS1 knockout. G) The interaction between LDHA and AARS1 was detected using Co‐IP in HEK293T cells transfected with pCMV‐Flag‐LDHA and pCMV‐HA‐AARS1. H) Co‐IP analysis of the interaction between exogenous pCMV‐HA‐AARS1 and endogenous LDHA in A549 cells. I) Co‐IP analysis of the interaction between endogenous AARS1 and endogenous LDHA in A549 cells. J) The interaction between GST‐LDHA and AARS1 was examined in vitro using a GST pull‐down assay. K) Immunofluorescence staining and co‐localization analysis of LDHA and AARS1 in A549 cells. L) Lactylation of LDHA in A549 cells overexpressing control vector, AARS1, or its 5 M mutant. M) Lactylation of LDHA and its 2KR mutant in A549 cells overexpressing AARS1. N) Activity assay of immunopurified LDHA proteins co‐incubated with AARS1‐WT/AARS1‐5 M in lactate‐containing buffer. O,P) The clonogenic and proliferative capacities (P) of A549 cells with LDHA knockdown and overexpression of either LDHA‐WT or LDHA‐2KR were evaluated in the context of AARS1 expression. Data are presented as mean ± SD from three independent experiments. ^*^ *p* < 0.05, ^**^ *p* < 0.01, ^***^ *p* < 0.001; ns stands for no significant change.

AARS1, a recently identified and potent lactyltransferase localized in the cytoplasm, has been shown to mediate lactylation modifications on a wide range of downstream target proteins.^[^ ^9^, ^31^ ^]^ To investigate the impact of AARS1 on LDHA lactylation, HA‐AARS1 and Flag‐LDHA were co‐transfected into A549 cells. Immunoprecipitation assays revealed a significant increase in lactylation modifications on LDHA, but the overexpression of AARS2 fails to exert a similar effect. (**Figure** **4**A; Figure S4C, Supporting Information). Furthermore, overexpression of AARS1 also markedly enhanced global protein lactylation levels in cells. (Figure 4B). To further validate the role of AARS1 in regulating LDHA lactylation, A549 cells with AARS1 knockout were generated using CRISPR/Cas9 technology. The lactylation of LDHA and global cellular proteins were then assessed. Knockout of AARS1 significantly suppressed both LDHA lactylation and global protein lactylation levels (Figure 4C,D). Furthermore, we found that AARS1 does not affect LDHA protein expression (Figure S4D, Supporting Information). Knockout of AARS1 also resulted in a significant reduction in the proliferative capacity and colony formation ability of A549 cells (Figure 4E,F), which is consistent with previous studies. Otherwise, Co‐IP analysis revealed a strong and specific association between exogenous Flag‐tagged LDHA and HA‐tagged AARS1 in HEK293T cells (Figure 4G). This finding was further validated in A549 cells, wherein both endogenous and exogenous AARS1 exhibited binding capability to the LDHA protein. (Figure 4H,I). Immunofluorescence results also demonstrated the co‐localization of LDHA and AARS1 in A549 cells (Figure 4I). To verify whether LDHA directly binds to AARS1 in vitro, GST‐LDHA was induced to express via the *Escherichia coli* expression system and purified using a glutathione affinity column for the GST‐pulldown assay. The GST‐pulldown assay demonstrated that LDHA can directly bind to AARS1 (Figure 4J), further validating the aforementioned findings.

Previous studies have demonstrated that mutations in the amino acid residues within the catalytic pocket of AARS1 (R77A, M100A, W176E, V218D, D239A) disrupt its interaction with lactate. The AARS1‐5 M mutant completely loses its lactyltransferase activity in vitro.^[^ ^31^ ^]^ To determine whether the 5 M mutation in AARS1 affects its other protein functions, we compared the subcellular localization and protein degradation rates between AARS1‐WT and AARS1‐5 M in A549 cells. The results showed that the AARS1‐5 M mutation does not influence the protein localization or degradation of AARS1 (Figure S4E,F, Supporting Information). To further validate that AARS1 was the lactyltransferase for LDHA, HA‐AARS1‐WT and HA‐AARS1‐5 M were co‐transfected with Flag‐LDHA into HEK293T cells, followed by treatment with NaLa. As expected, AARS1‐WT significantly enhanced LDHA lactylation under conditions of abundant lactate substrate, whereas the lactyltransferase function of AARS1‐5 M toward LDHA was abolished (Figure 4J). Furthermore, we observed that AARS1 significantly enhanced the lactylation of LDHA‐WT but failed to increase the lactylation of LDHA‐2KR (Figure 4K), further validating the role of AARS1 in regulating LDHA lactylation. Immunopurified LDHA‐WT or LDHA‐2KR proteins were co‐incubated with lysates from HEK293T cells overexpressing AARS1‐WT or AARS1‐5 M in lactate‐containing buffer. Subsequent activity assays revealed that AARS1‐WT significantly enhanced the enzymatic activity of LDHA‐WT (but not LDHA‐2KR), while AARS1‐5 M failed to produce this stimulatory effect (Figure 4L). Our results demonstrate that AARS1, as the lactyltransferase for LDHA, plays a critical role in regulating the enzymatic activity of LDHA. To determine whether lactylation of LDHA affects the composition of tetramers (LDH1‐LDH5), we performed native gel electrophoresis to analyze LDH isoenzymes. The results showed that AARS1 knockout had no effect on the composition of LDH tetramers. (Figure S4G, Supporting Information). Then we explored the oncogenic function of AARS1 through modulating LDHA lactylation. In A549 cells with LDHA knockdown and overexpression of LDHA‐WT, AARS1 overexpression significantly enhanced clonogenic ability and proliferation. However, this effect was not observed in cells with LDHA knockdown and overexpression of the lactylation‐defective mutant LDHA‐2KR (Figure 4O,P). These findings indicate that AARS1 may promote tumor progression by regulating LDHA lactylation.

### 2.5 SIRT2 is the Potential Eraser of LDHA Lactylation in LUAD Cells

**Figure 5:**
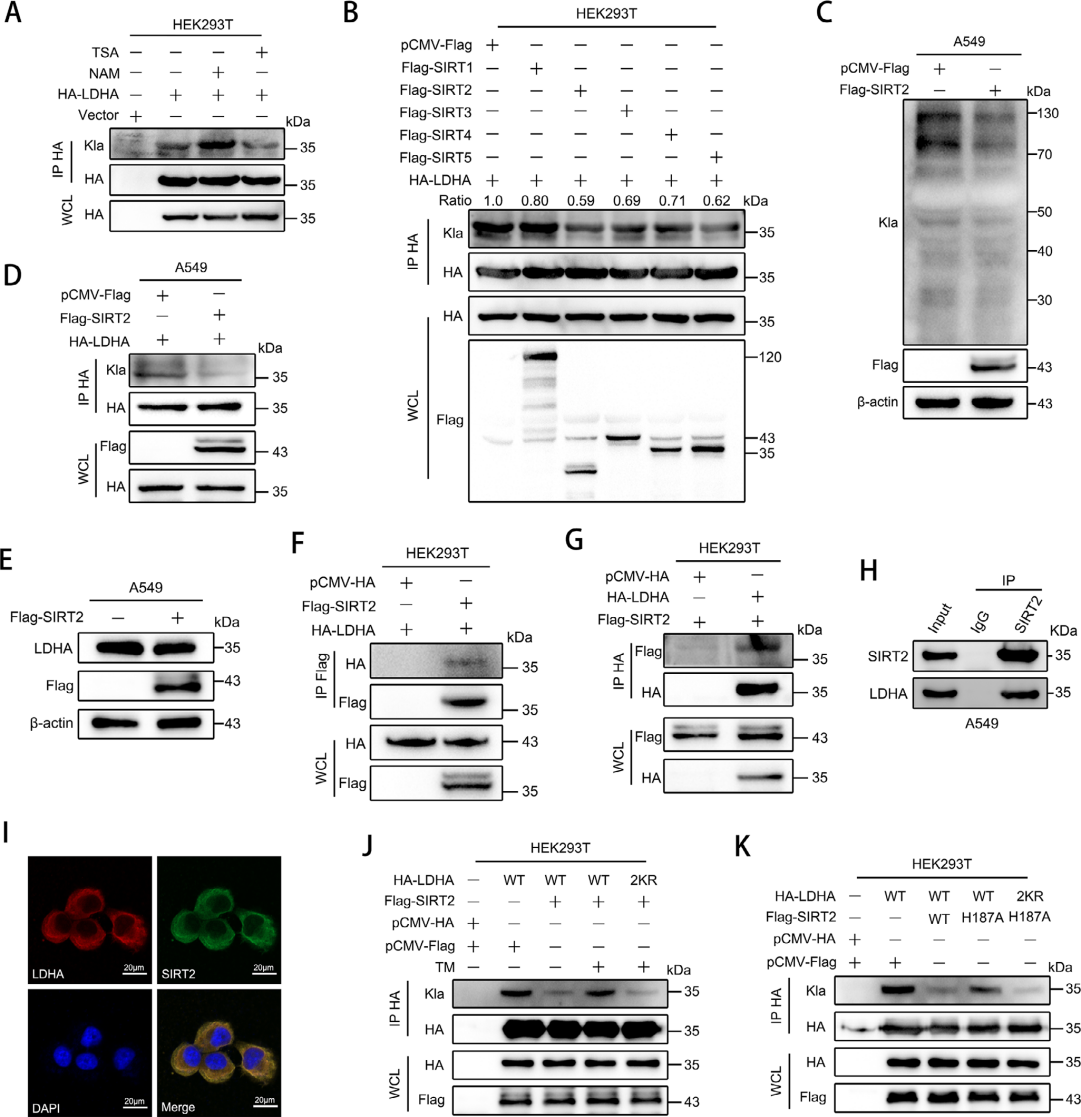
SIRT2 is the potential eraser of LDHA lactylation in LUAD cells. A) Lactylation levels of LDHA in LDHA‐overexpressing cells treated with TSA (0.5 µm) or NAM (5 mm). B) Effects of overexpressing cytosolic SIRT family proteins (SIRT1‐SIRT5) on LDHA lactylation. C) Global lactylation levels in A549 cells overexpressing SIRT2. D) Lactylation of LDHA in SIRT2‐overexpressing A549 cells. E) Detection of LDHA protein levels in SIRT2‐overexpressing A549 cells. F,G) Interaction between SIRT2 and LDHA in 293T cells co‐overexpressing both proteins. H) Co‐IP analysis of the interaction between endogenous SIRT2 and endogenous LDHA in A549 cells. I) Immunofluorescence co‐localization of SIRT2 and LDHA in A549 cells. J) Lactylation analysis of HA‐LDHA‐WT and HA‐LDHA‐2KR in HEK293T cells co‐transfected with SIRT2, with or without 10 µm TM treatment for 24 h. K) Lactylation analysis of HA‐LDHA‐WT and HA‐LDHA‐2KR in HEK293T cells co‐transfected with SIRT2‐WT or SIRT2‐H187A.

To probe possible enzymes responsible for the delactylation of LDHA, HEK293T cells transfected with LDHA were treated with the deacetylase inhibitor trichostatin A (TSA) or nicotinamide (NAM). The results demonstrated that NAM treatment significantly increased the lactylation levels of LDHA (**Figure** **5**A). To further identify the specific enzyme responsible for LDHA delactylation, we conducted a lactylation screen in LDHA‐overexpressing HEK293T cells cotransfected with individual members of the sirtuin family of deacetylases (SIRT1–SIRT5), which localized in the cytoplasm. The results demonstrated that overexpression of SIRT2, but not other members of the SIRT family, significantly suppressed the lactylation of LDHA (Figure 5B). Furthermore, overexpression of SIRT2 also strongly suppressed global lactylation levels in A549 cells (Figure 5C), indicating that SIRT2 indeed functions as a delactylase to regulate cellular lactylation. Notably, SIRT2 overexpression significantly suppressed LDHA lactylation without affecting its protein expression levels in A549 cells (Figure 5D,E). Meanwhile, co‐immunoprecipitation assays revealed an interaction between SIRT2 and LDHA (Figure 5F–H), and immunofluorescence results demonstrated that SIRT2 co‐localized with LDHA in the cytoplasm (Figure 5I). To investigate whether the delactylase function of SIRT2 depends on its deacetylase activity, we co‐transfected HEK293T cells with HA‐LDHA and Flag‐SIRT2, followed by treatment with 10 µm of the SIRT2‐specific inhibitor thiomyristoyl (TM). Immunoprecipitates revealed that while SIRT2 normally significantly suppresses LDHA lactylation, TM treatment markedly attenuated this inhibitory effect (Figure 5J). Furthermore, we generated a deacetylase‐deficient SIRT2 mutant (SIRT2‐H187A) and confirmed that the H187A mutation does not affect the subcellular localization or degradation rate of SIRT2 (Figure S5A,B, Supporting Information). Subsequently, we evaluated its delactylation activity toward LDHA. Compared to SIRT2‐WT, the SIRT2‐H187A mutant exhibited a significantly reduced ability to delactylate LDHA (Figure 5K). Collectively, our data establish SIRT2 as a bona fide LDHA delactylase whose delactylase function depends on deacetylase activity.

### 2.6 LDHA Affects the DNA Damage Response Induced by Cisplatin by Regulating the Overall Lactate Level of LUAD Cells

**Figure 6:**
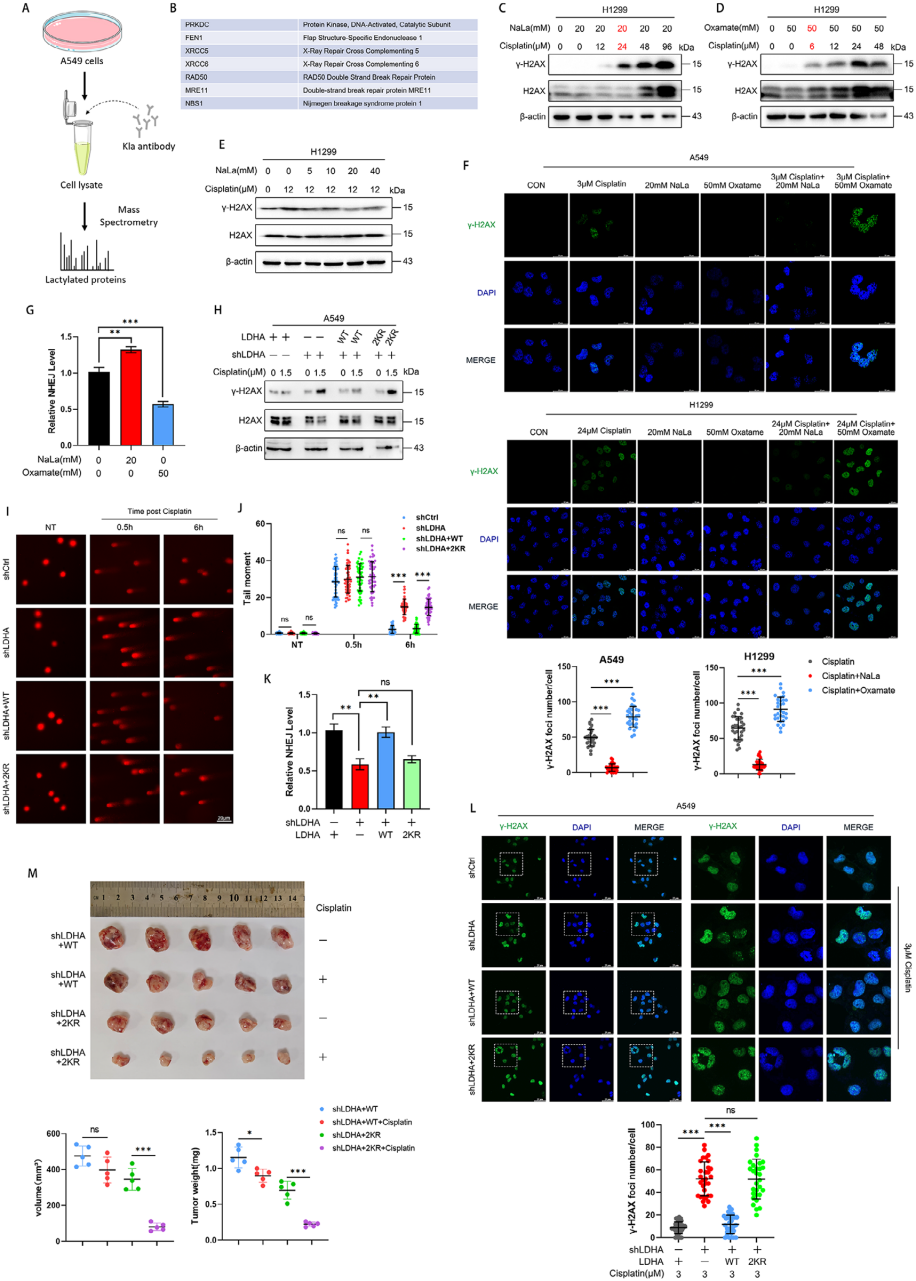
LDHA affects the DNA damage response induced by cisplatin by regulating the overall lactate level of LUAD cells. A) Mass spectrometry schematic for identifying lactylated peptides using anti‐Lactyl‐lysine. B) Proteins related to DNA damage repair were screened by lactated modified mass spectrometry. C) H1299 cells were treated with 20 mm NaLa (24 h) and concentration gradient cisplatin (12 h), after which the cells were lysed, and protein immunoblotting was performed to detect DNA damage. D) The H1299 cells were treated with 50 mm Oxamate (24 h) and concentration gradient cisplatin (12 h), immunoblotting was performed to detect the DNA damage. E) H1299 cells were treated with concentration gradient NaLa (24 h) and 12 µm cisplatin (12 h), immunoblotting was performed to detect DNA damage. F) After the cells were treated with cisplatin, NaLa and Oxamate, the cells were stained with DAPI and γ‐H2AX at a scale of 25 µm. G) The NHEJ level in the indicated A549 cells was assessed by FACS. H) The LDHA‐WT and LDHA‐2KR plasmids were overexpressed in the shLDHA ‐A549 stable cell line, treated with 1.5 µm cisplatin (12 h), lysed the cells, and performed protein immunoblotting to detect DNA damage. I,J) Representative (I) and quantification (J) graphs of tail moments in A549 cells with LDHA knockdown followed by reconstitution with either wild‐type (WT) or 2KR mutant. NT, no treatment. K) The NHEJ level in indicated A549 cells was assessed by FACS. L) Overexpression of LDHA‐WT and LDHA‐2KR plasmid in the shLDHA ‐A549 stable cell line was performed by DAPI and γ‐H2AX staining. M) LLC cells with LDHA knockdown followed by reconstitution with either wild‐type (WT) or the 2KR mutant were subcutaneously injected into C57BL/6J mice. After tumor formation, the mice either received no treatment or were treated with cisplatin (2 mg kg^−1^ every 2 days) via intraperitoneal injection. Tumor images were acquired, and tumor volume and weight were measured. Data are presented as mean ± SD from three independent experiments. ^*^ *p* < 0.05, ^**^ *p* < 0.01, ^***^ *p* < 0.001; ns stands for no significant change.

In order to further explore the effect of LDHA on the lactylation of proteins by affecting the intracellular lactate level, we used lactylation specific antibodies combined with co‐immunoprecipitation and mass spectrometry (MS) to identify the proteins that may be lactylated (**Figure** **6**A; Table S2, Supporting Information). Through MS analysis, we identified five DNA damage repair proteins involved in NHEJ. Furthermore, we detected other DNA damage repair proteins—such as MRE11 and NBS1, which are known to be regulated by lactylation (Figure 6B).^[^ ^10^, ^20^ ^]^ This finding further corroborates the reliability of the mass spectrometry data and suggests that LDHA might influence the DNA damage repair process by modulating overall cellular lactate levels and lactylation. In order to explore the specific mechanism, we used cisplatin to construct DNA damage models in A549 and H1299 cells (Figure S6A, Supporting Information), and we also excluded the effect of Oxamate on DNA damage (Figure S6B, Supporting Information). By using NaLa and Oxamate to change the lactylation level of cells, we found that NaLa significantly increased the cisplatin concentration required to induce DNA damage (Figure 6C; Figure S6C, Supporting Information), while Oxamate had the opposite effect (Figure 6D; Figure S6D, Supporting Information), and NaLa significantly inhibited the DNA damage (Figure 6E; Figure S6E, Supporting Information). Meanwhile, we also verified these results through immunofluorescence experiments using the puncta of γ‐H2AX as the indicator. The results showed that NaLa alleviated the DNA damage, while Oxamate promoted this process (Figure 6F). Oxamate also promoted the cisplatin‐induced cell death (Figure S6F, Supporting Information). Further analysis revealed that NaLa enhanced the efficiency of NHEJ in cisplatin‐treated cells, whereas Oxamate attenuated it (Figure 6G; Figure S6G, Supporting Information). We also found that after LDHA knockdown in A549 cells, the cisplatin concentration required for DNA damage was significantly reduced, while the required cisplatin concentration was significantly increased after LDHA‐WT was re‐expressed, and the required cisplatin concentration was not affected after LDHA‐2KR was overexpressed (Figure S6H, Supporting Information). Western Blot and immunofluorescence experiment also confirmed the above results (Figure 6H,L). To investigate whether the lactylation level of LDHA played a critical role in DNA damage repair, the average tail moment of cells was assessed after cisplatin treatment (Figure 6I,J). The results demonstrated that deficiency in LDHA lactylation significantly impaired DNA damage repair efficiency. Consistent with this finding, NHEJ efficiency was also reduced (Figure 6K; Figure S6I, Supporting Information). To investigate whether lactylation of LDH influences sensitivity to cisplatin therapy, LLC cells were subcutaneously inoculated into C57BL/6J mice to generate tumor xenografts, which were then treated with low‐dose cisplatin. The results indicated that LLC cells with normal LDH lactylation were significantly less sensitive to low‐dose cisplatin compared to those with deficient LDH lactylation (Figure 6M). In summary, our results suggest that LDHA influences the DNA damage response by regulating the overall lactate level of LUAD cells.

### 2.7 LDHA Affects DNA Damage Repair by Regulating Global Lactylation of LUAD Cells

**Figure 7:**
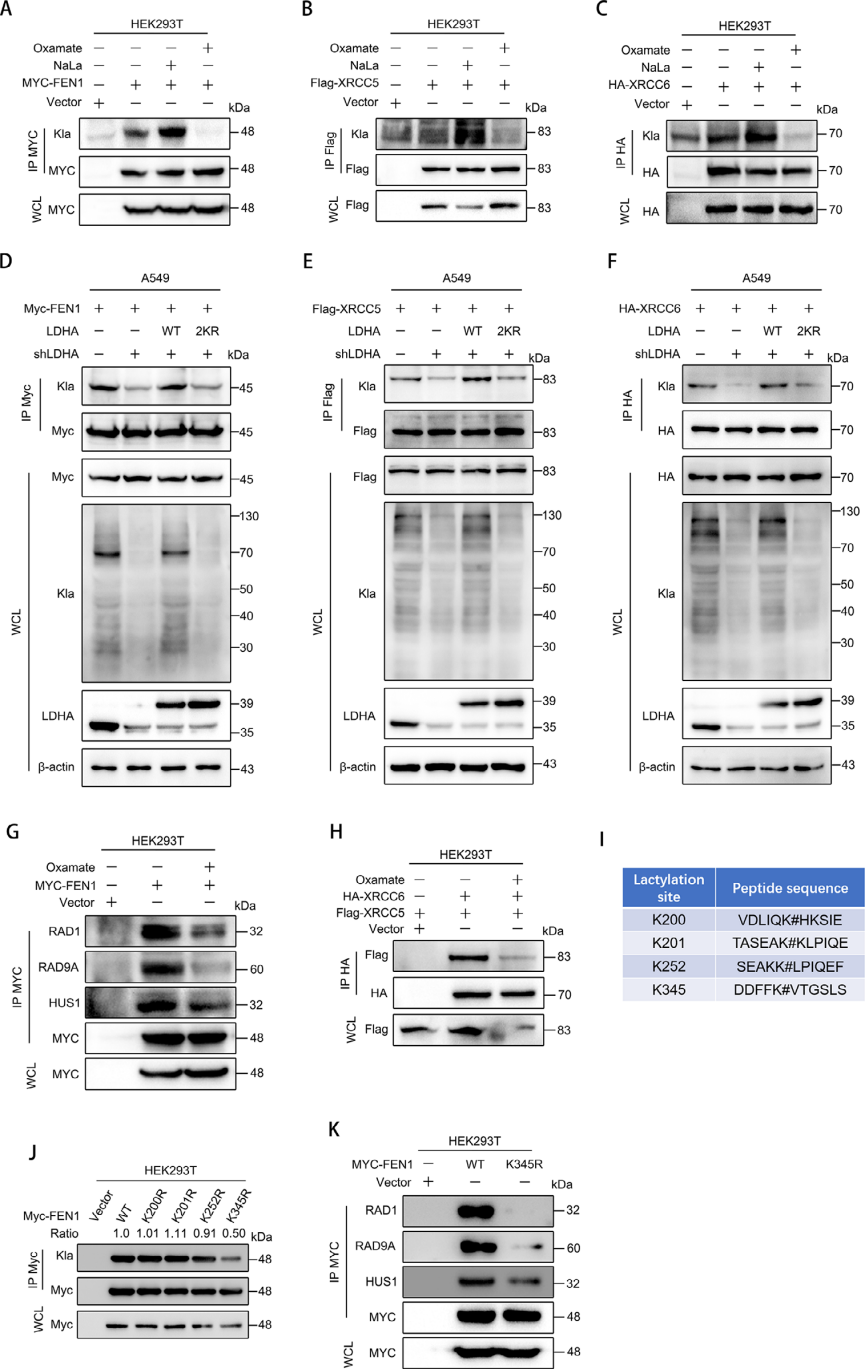
LDHA affects DNA damage repair by regulating global lactylation of LUAD cells. A–C) FEN1, XRCC5, and XRCC6 were overexpressed in 293T cells, and the cells were treated with NaLa (20 mm) and Oxamate (50 mm) for 24 h. Whole cell extracts were collected for immunoprecipitation assay, and then protein immunoblot assay was performed. D–F) FEN1, XRCC5, and XRCC6, respectively, were overexpressed with LDHA‐WT and LDHA‐2KR plasmid in the shLDHA‐A549 stable cell line. Whole cell extracts were collected for immunoprecipitation assay, and then protein immunoblotting assay was performed. G) FEN1 plasmid was overexpressed in 293T cells and Oxamate (50 mm) was treated with the cells for 24 h. Whole cell extracts were collected for co‐immunoprecipitation assay, and then protein immunoblot assay was performed. H) Overexpression of XRCC5 and XRCC6 plasmid in 293T cells was combined with treatment of Oxamate (50 mm) cells for 24 h, whole cell extracts were collected for immunoprecipitation assay, and then protein immunoblot assay was performed. I) FEN1 lactylation sites through integrated analysis of published lactylome datasets. J) Screening of lactylation sites in FEN1. K) Analysis of the interaction between FEN1‐WT/K345R and DNA damage repair adaptor molecules (RAD1, RAD9A, and HUS1).

**Figure 8:**
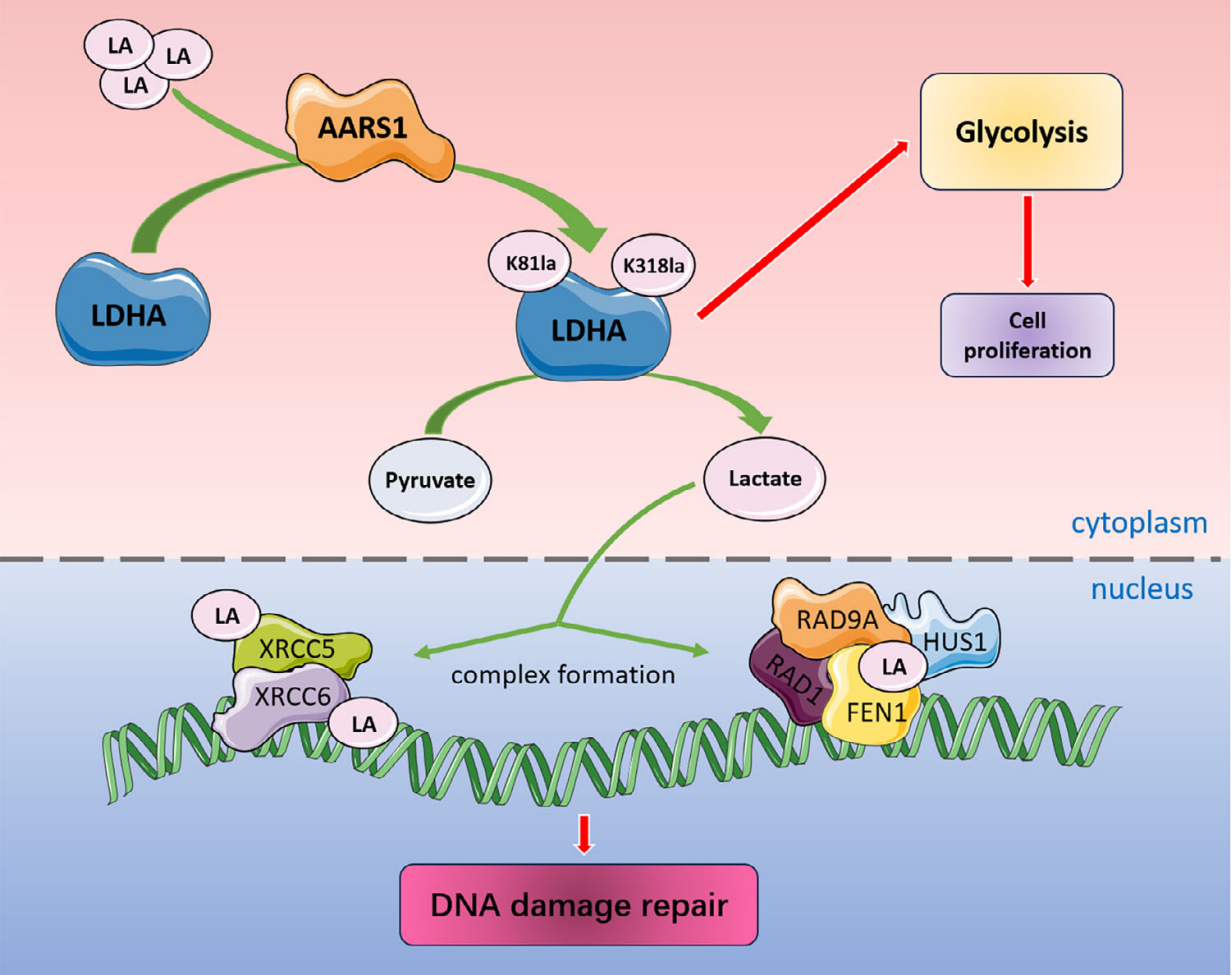
Graphic model as discussed in the text. Bidirectional regulation between LDHA and lactylation enhances glycolysis and DNA damage repair.

We then verified the results of mass spectrometry by detecting the lactylation of FEN1, XRCC5, and XRCC6 proteins (**Figure** **7**A–C). Additionally, MRE11 and NBS1, which have been previously confirmed to be regulated by lactylation, were also validated (Figure S7A,B, Supporting Information). We also found that after LDHA was knocked down in A549 cells, the lactylation levels of these three proteins were significantly reduced, while the lactylation of these three proteins were significantly increased after the re‐expression of LDHA‐WT, but the overexpression of LDHA‐2KR did not show this effect. The global lactylation also showed the same trend (Figure 7D–F). These suggest that LDHA affects the lactylation level of proteins involved in DNA damage repair. In order to further explore whether lactylation of these proteins affects the DNA damage repair function, we treated cells with Oxamate and found that Oxamate significantly inhibited the formation of FEN1‐RAD1‐RAD9A‐HUS1, the necessary four‐element complex for NHEJ (Figure 7G). At the same time, Oxamate also significantly inhibited the formation of XRCC5‐XRCC6, a key binary complex of nonhomologous end connection repair mode (Figure 7H). Existing lactylome data indicate that K200, K201, K252, and K345 are the primary lactylation sites on FEN1 (Figure 7I).^[^ ^27^ ^]^ Through subsequent screening, we found that the K345R mutation significantly reduces lactylation of FEN1(Figure 7J). Further studies revealed that, compared to FEN1‐WT, the interaction between FEN1‐K345R and DNA damage repair adaptor molecules such as RAD1, RAD9A, and HUS1 was markedly diminished—a phenomenon highly consistent with the effects observed after Oxamate treatment (Figure 7K). This suggests that LDHA affects NHEJ by regulating the lactylation of the proteins involved in this process (**Figure** **8**).

## 3 Discussion

As a recently discovered new type of post‐translational modification, lactylation has received great attention in the fields of metabolic reprogramming and disease regulation. By modulating gene expression and protein function, lactylation influences various disease progression, and previous studies have demonstrated its role in the development of various cancers, including gastric cancer,^[^ ^32^ ^]^ pancreatic cancer,^[^ ^11^ ^]^ and melanoma.^[^ ^33^ ^]^ Inhibition of lactylation has been demonstrated to attenuate tumor progression in LUAD.^[^ ^34^, ^35^ ^]^ The regulatory mechanisms governing cellular lactylation are remarkably complex. While p300 mediates histone lactylation using lactyl‐CoA as a substrate,^[^ ^36^ ^]^ both AARS1 and AARS2 directly utilize lactate molecules to catalyze lactylation of cytoplasmic and mitochondrial proteins.^[^ ^7^, ^37^ ^]^ Notably, LDHA—the key enzyme responsible for lactate production—has been demonstrated to regulate global lactylation levels through controlling the intracellular lactate concentration.^[^ ^38^, ^39^ ^]^ In this study, we found significantly enhanced lactylation in LUAD tissues compared to normal lung tissues. Mechanistically, we discovered that LDHA itself underwent lactylation, which enhanced its activity through a positive feedback loop. Furthermore, LDHA regulated DNA damage repair by modulating the lactylation of key NHEJ proteins, such as FEN1, XRCC5, and XRCC6. We revealed a bidirectional regulatory mechanism between LDHA and lactylation: lactylation enhances LDHA activity, promoting the catalytic production of more lactate and mediating global protein lactylation. This discovery elucidates a previously unrecognized mechanistic link between lactylation‐mediated metabolic reprogramming and the Warburg effect in tumorigenesis.

Lactate dehydrogenase (LDH) exists as homotetramers or heterotetramers composed of LDHA and LDHB subunits in varying stoichiometric ratios. The distinct substrate affinities of these two subunits enable precise regulation of the reversible pyruvate‐lactate interconversion.^[^ ^14^ ^]^ LDHA exhibits a more pronounced role than LDHB in promoting the Warburg effect,^[^ ^40^ ^]^ likely attributable to structural differences between these isoforms that influence their respective substrate affinities.^[^ ^41^ ^]^ Our Western blot analyses at both tissue and cellular levels demonstrated that LDHA exhibits a stronger association with lactylation compared to LDHB. Notably, knockdown of LDHA, but not LDHB, led to global downregulation of cellular lactylation. Furthermore, lactate supplementation rescued the proliferation inhibition caused by LDHA depletion, suggesting that LDHA's oncogenic function may be mediated through regulation of cellular lactylation. These findings corroborate previous work by Li et al, who showed that LDHA knockdown suppresses pancreatic ductal adenocarcinoma proliferation both in vitro and in vivo by inhibiting histone H3K18 lactylation.^[^ ^42^ ^]^ Collectively, this compelling evidence establishes LDHA as a master regulator of cellular lactylation dynamics.

LDHA as a critical mediator of oncogenesis through its master regulation of the Warburg effect exhibits markedly elevated activity in malignant tissues. While the central role of LDHA in governing cellular lactylation is well‐documented in the literature, whether LDHA itself undergoes lactylation remains unclear. Our study first discovered that LDHA itself underwent lactylation with K81 and K318 identified as critical lactylation sites. Succinylation at the K222 residue of LDHA has been shown to attenuate its K63‐linked polyubiquitination and subsequent degradation by impairing interaction with the selective autophagy receptor SQSTM1/p62.^[^ ^17^ ^]^ To investigate whether lactylation at K81 and K318 similarly affects protein turnover, we performed comparative degradation assays. Notably, the degradation kinetics of LDHA K81R/K318R mutants remained comparable to wild‐type LDHA, indicating that these lactylation sites did not significantly influence protein stability. A recent groundbreaking study demonstrated that lysine acetyltransferase 7 (KAT7) enhances both LDHA enzymatic activity and protein expression through acetylation of lysine 118 (K118) on LDHA.^[^ ^43^ ^]^ Furthermore, Chen et al. demonstrated that zinc finger DHHC‐type palmitoyltransferase 9 (ZDHHC9)‐mediated palmitoylation serves as a positive regulator of LDHA, enhancing its enzymatic activity through increased palmitoylation and consequently conferring chemoresistance in pancreatic adenocarcinoma.^[^ ^16^ ^]^ These paradigm‐shifting findings collectively establish post‐translational modifications as critical determinants of LDHA activity. Building upon prior experimental evidence, we postulated that lactylation might regulate LDHA enzymatic activity – a mechanistic relationship that had not been previously documented. To validate this hypothesis, we conducted comparative in vitro activity assays between LDHA mutants and wild‐type protein. Our results demonstrated that ablation of lactylation at residues K81 and K318 significantly impaired LDHA catalytic function, as further corroborated by metabolic profiling showing reduced pyruvate‐to‐lactate conversion and diminished ATP production. These findings reveal a potential positive feedback loop in tumors where elevated lactate levels may amplify the Warburg effect through lactylation‐mediated enhancement of LDHA activity.

Substantial experimental evidence has delineated a strong functional link between protein lactylation and DNA damage repair mechanisms. Chen et al. demonstrated that lactylation of NBS1 at lysine 388 (K388) was essential for both formation of the MRE11‐RAD50‐NBS1 (MRN) complex and the recruitment of homologous recombination (HR) repair proteins to DNA double‐strand break sites.^[^ ^10^ ^]^ Additional research findings suggested that XRCC1 underwent lactylation at lysine 247 (K247) and lactylated XRCC1 showed a stronger affinity for importin α, allowing for greater nuclear transposition of XRCC1 and enhanced DNA damage repair.^[^ ^44^ ^]^ However, whether lactylation participated in the NHEJ repair pathway remains unclear. Previous studies have demonstrated that methylation at Arg192 of FEN1 suppressed its phosphorylation at Ser187. The methylated form of FEN1 maintained interaction with proliferating cell nuclear antigen (PCNA), Mutations of FEN1 disrupted arginine methylation and led to a defect in Okazaki fragment maturation, a delay in cell cycle progression, impairing DNA repair and a high frequency of genome‐wide mutations.^[^ ^45^ ^]^ Chen et al. proposed that PORCN, through its S‐acyltransferase activity, mediated S‐palmitoylation at five specific cysteine residues of XRCC6, playing a significant role in NHEJ repair.^[^ ^46^ ^]^ Our findings revealed that key NHEJ repair proteins, including FEN1, XRCC5, and XRCC6, underwent lactylation. We demonstrated that lactylation was essential for the proper assembly of both FEN1‐RAD1‐RAD9A‐HUS1 complex and the XRCC5‐XRCC6 complex, as evidenced by significantly impaired protein interactions under low‐lactylation conditions. Bhatt et al. have proved that Enhanced glycolytic flux promoted the rejoining of radiation‐induced DNA strand breaks through activation of both NHEJ and homologous recombination (HR) repair pathways, ultimately reducing radiation‐induced cytogenetic damage (micronucleus formation) in these cells.^[^ ^47^ ^]^ Thus, We provide definitive evidence for a sophisticated reciprocal regulation between glycolytic metabolism and DNA repair machinery.

Taken together, our study has comprehensively characterized the mechanistic basis of LDHA lactylation and elucidated its role in promoting LUAD progression. Besides, we also demonstrated that the therapeutic resistance to cisplatin of LUAD was mediated by the activation of NHEJ via lactylation modifications. Our findings reveal an intricate bidirectional regulatory network where lactylation is both regulated by and functionally modulates LDHA, establishing a previously unrecognized positive feedback loop between LDHA activity and global protein lactylation. These insights significantly advance our understanding of lactylation‐mediated regulatory mechanisms in cancer biology.

## 4 Experimental Section

### Antibodies and Reagents

Antibodies: Kla (1:1000, PTM BIO Cat# PTM‐1401RM, RRID:AB_2 942 013), LDHA (1:2000, Proteintech Cat# 19987‐1‐AP, RRID:AB_10 646 429), LDHB (1:2000, Proteintech Cat# 14824‐1‐AP, RRID:AB_2 134 953), p300 (1:1000, Proteintech Cat# 20695‐1‐AP, RRID:AB_3 085 614) AARS1 (1:1000, Proteintech Cat# 67909‐1‐Ig, RRID:AB_2 918 664), SIRT2 (1:1000, Proteintech Cat# 66410‐1‐Ig, RRID:AB_2 881 782), HA (1:1000, Thermo Fisher Scientific Cat# 26183‐D550, RRID:AB_2 533 052), Flag (1:1000, Proteintech Cat# 66008‐4‐Ig, RRID:AB_2 918 475), MYC (1:1000, Proteintech Cat# 60003‐2‐Ig, RRID:AB_2 734 122), H2A.X (1:1000, Proteintech Cat# 10856‐1‐AP, RRID:AB_2 114 985), γ‐H2AX (1:1000, Proteintech Cat# 29380‐1‐AP, RRID:AB_3 085 345) and β‐actin (1:3000, Proteintech Cat# 60008‐1‐Ig, RRID:AB_2 289 225).

Sodium oxamate (S123221) and Sodium lactate (S108838) were ordered from Aladdin. MG132 (C1791) was purchased from Biovision. Polyformaldehyde (P1110, 4%) was purchased from Solarbio. Cisplatin (HY‐17394) was purchased from MCE.

### Cell Culture and Transfection

The human lung epithelial cell line BEAS‐2B (RRID:CVCL_0168) was purchased from the National Collection of Authenticated Cell Cultures and cultured in RPMI 1640 (Gibco) supplemented with 10% FBS (SORFA), LUAD cell lines (A549 (RRID:CVCL_A549), H1299 (RRID:CVCL_XE62), H23 (RRID:CVCL_1547), HCC827 (RRID:CVCL_2063), H358 (RRID:CVCL_1559) and PC9 (RRID:CVCL_B7N9)) and mouse Lewis lung carcinoma cells (LLC) (RRID:CVCL_4358) were purchased from the cell library of the Chinese Academy of Sciences were cultured in RPMI 1640 (Gibco) supplemented with 10% FBS (SORFA). HEK293T (RRID:CVCL_0063) cells were purchased from the National Collection of Authenticated Cell Cultures and cultured in DMEM (Dulbecco's Modified Eagle Medium, Gibco) supplemented with 10% FBS (SORFA). All cells were cultured under an atmosphere of 5% CO2 at 37 °C. All cell lines used in this study were acquired by laboratory in December 2021. Cells were routinely tested for mycoplasma contamination with negative results and all experiments were conducted within 10 passages from original stocks to ensure genetic stability.

Cells at 70–80% confluence were transfected with the indicated plasmids using the SuperFectin DNA Transfection Reagent kit (Pufei, 2103–100). siRNA transfection was performed with siTran 2.0 siRNA transfection reagent (OriGene, TT320002) when cell confluence reached 50%. All transfection experiments were performed according to the instruction manual.

### Immunoprecipitation Assay

Following three PBS washes, cells were lysed in appropriate volumes of PMSF‐containing lysis buffer (0.5% Nonidet P‐40, 150 mm NaCl, 1 mm sodium orthovanadate, 20 mm β‐glycerophosphate, 20 mm NaF, 20 mm HEPES, pH 7.4) at 4 °C for 30 min. After centrifugation, the supernatants were incubated with antibodies and Protein G‐agarose beads (Roche, 11 243 233 001) at 4 °C for 12 h. The beads were then pelleted, washed three times with 0.5% Nonidet P‐40, and resuspended in 2 × loading buffer followed by boiling for 10 min.

### Western Blotting

Cells were washed three times with ice‐cold PBS and lysed in chilled buffer (0.5% Nonidet P‐40, 150 mm NaCl, 1 mm sodium orthovanadate, 20 mm β‐glycerophosphate, 20 mm NaF, 20 mm HEPES) at 4 °C for 30 min. Following centrifugation at 12 000 × g for 15 min, total protein concentration was quantified using a BCA Protein Assay Kit (Thermo Fisher Scientific). Equal amounts of protein from each group were resolved by SDS‐PAGE and electrophoretically transferred onto PVDF membranes (Millipore, IPVH00010) at 120 V for 2 h. Membranes were blocked with 5% nonfat dry milk (Solarbio, D8340) in TBST for 2 h at room temperature, then incubated with primary antibodies overnight at 4 °C, followed by HRP‐conjugated secondary antibodies. Protein signals were detected using ECL substrate (TIANGEN, PA112‐01) and visualized through digital gel imaging (TANON 5500).

### Cell Growth Assay

Cell suspensions containing 3–5 × 10^3^ cells in RPMI 1640 medium supplemented with 10% fetal bovine serum were plated into 24‐well plates, with triplicate wells for each experimental condition. Following the designated incubation period, cellular fixation was performed using 4% formaldehyde solution at ambient temperature for 15 min, after which cells were stained with 1% crystal violet. Subsequent incubation with 10% acetic acid enabled quantification of relative proliferation through spectrophotometric measurement at 595 nm.

### For the Colony Formation Assay

Cell suspensions containing 500 cells in 1 mL of complete medium (10% FBS supplementation) were plated into 6‐well culture plates. Culture media were refreshed at 48‐h intervals. Following 10 days of incubation, cellular colonies were fixed with 4% formaldehyde (30 min) and visualized using 0.1% crystal violet staining. Colony images were captured with a Canon EOS70D digital imaging system.

### Transwell Migration Assay and Scratch Wound Healing Assay

Cell migration was assessed using Transwell chambers (8 µm pores). Tumor cells (5 × 10^4^–1 × 10^5^) in 200 µL serum‐reduced medium (RPMI 1640 with 1% FBS) were loaded into the upper chamber, while the lower compartment contained 500 µL chemoattractant medium (RPMI 1640 with 10% FBS). Following incubation, migrated cells on the membrane underside were fixed (4% formaldehyde) and stained (1% crystal violet) for quantification using an Olympus IX71 microscope. For wound healing assays, confluent monolayers (80–90%) in 6‐well plates were scratched with sterile pipette tips (200 µL). After PBS washing (3 ×), low‐serum medium (1% FBS) was added. Migration was monitored by phase‐contrast microscopy at designated intervals.

### LDHA Knockdown and Reintroduction

For LDHA gene reconstitution, A549, H1299, and LLC cells with stable LDHA knockdown were sequentially transduced with lentiviral vectors expressing human FLAG‐tagged LDHA‐WT, LDHA‐K81R, LDHA‐K318R, and LDHA‐2KR constructs. Polybrene (8 µg mL^−1^) was added to enhance transduction efficiency. sub‐confluent cultures of each cell line were infected with 3 mL viral supernatant for 24 h, followed by puromycin selection (A549: 2 µg mL^−1^; H1299: 3 µg mL^−1^; LLC: 1.5 µg mL^−1^) for 1 week to establish stable transductants.

### CRISPR‐Cas9‐Mediated Gene Knockout

Targeting sgRNA sequences for AARS1 were designed using the CHOPCHOP web tool (https://chopchop.cbu.uib.no/). The designed sgRNAs were subsequently cloned into the lentiCRISPR‐v2‐Flag vector. HEK293T cells were seeded in 6‐well plates at a density of 5 × 10⁵ cells per well and transfected at 90% confluence using Lipofectamine 3000 with the packaging plasmids pSPAX2 (2 µg), pMD2.G (1.5 µg), and the lentiCRISPR‐v2‐FLAG‐AARS1 construct (1 µg). Viral supernatants were collected 48 h post‐transfection, filtered through 0.45 µm membranes, and used to infect A549 cells. After 24 h infection, transduced cells were selected with 10 µg mL^−1^ puromycin (Solarbio, P8230). Single‐cell clones were isolated by limiting dilution in 96‐well plates and expanded. AARS1‐knockout (KO) stable cell lines were validated by Western blot analysis, with cells transfected with control CRISPR‐Cas9 plasmid serving as negative controls.

### Measurement of LDHA Enzyme Activity

LDHA‐WT‐Flag, LDHA‐K81R‐Flag, LDHA‐K318R‐Flag and LDHA‐2KR‐Flag were transfected into HEK293T cells and immunoprecipitated by Flag beads. Proteins were eluted by elution buffer [20 mm Hepes, 150 mm NaCl, 1 mm EDTA, 3*Flag peptide (200 µg mL^−1^) (pH 7.5)]. Purified LDHA proteins (1 µg) were added to reaction buffer (0.2 M Tris‐HCl, 30 mm pyruvate, and 2 mm NADH (pH 7.4)]. The absorbance (340 nm) was measured by spectrophotometric.

### GST Pull‐Down

The GST‐LDHA fusion protein expression system was constructed by inserting the LDHA coding fragment into the pGEX‐4T‐1 vector. Following the manufacturer's protocol, the GST‐LDHA fusion protein was purified using Pierce glutathione magnetic beads. Approximately 500 µg of lysate from HEK293T cells transfected with HA‐AARS1 was incubated with 2–5 µg of GST‐LDHA fusion protein in IP lysis buffer at 4 °C for 4 h. The GST‐LDHA fusion protein and its interacting partners were captured using Pierce glutathione magnetic beads. Bound proteins were released by heat denaturation in protein loading buffer. Subsequently, the samples were separated by SDS‐PAGE and analyzed via immunoblotting.

### LDH Isoenzyme Characterization

Five known lactate dehydrogenase (LDH) isoenzymes were separated and identified using native gel electrophoresis. A total of 20 µg of protein sample was loaded onto a 1.5% agarose gel, which was prepared in a buffer containing 25 mm Tris–HCl and 250 mm glycine (pH 9.5). The 6 × loading buffer consisted of 0.1% bromophenol blue, 15% glycerol, and 20 mm Tris–HCl (pH 8.0). Electrophoresis was performed at a constant voltage of 100 V for 150 min in a running buffer containing 5 mm Tris–HCl and 40 mm glycine (pH 9.5). After electrophoresis, the gel was briefly rinsed in 100 mm Tris–HCl buffer (pH 8.5). Subsequently, the gel was incubated at 37 °C for 40 min in a developing solution containing lactic acid (3.24 mg mL^−1^), β‐nicotinamide adenine dinucleotide (0.3 mg mL^−1^), nitrotetrazolium blue chloride (NBT, 0.8 mg mL^−1^), and phenazine methosulfate (PMS, 0.167 mg mL^−1^) prepared in 10 mm Tris–HCl buffer (pH 8.5).

### Allograft

C57BL/6J (RRID:IMSR_JAX:000664) mice were obtained from GemPharmatech (Jiangsu, China) and maintained under specific pathogen‐free (SPF) conditions at the Laboratory Animal Center of Nanchang University. All experimental procedures were approved by the Institutional Animal Care and Use Committee of Nanchang University (Approval No. NCULAE‐20250418002) and conducted in strict accordance with established ethical guidelines for animal research.

For tumor allograft studies, LLC cells (1 × 10^5^) stably expressing the control plKO.1 plasmid or LDHA knockdown or LDHA‐knockdown LLC cells rescued with LDHA‐WT or LDHA‐2KR were injected subcutaneously into the anterior axilla of 5‐week‐old female C57BL/6J mice. Tumors were harvested at day 20 post‐injection, with volumes calculated by ellipsoid formula (calculated as 𝜋/6 × [large diameter] × [smaller diameter]^2^) and weights measured.

### Immunofluorescent Staining

A total of 1000 cells were seeded onto cell slides (NEST 801 010) placed in 24‐well plates and cultured for 24 h. The cell slides were washed three times with PBS, fixed with absolute ethanol for 30 min, and subsequently rinsed with PBS to remove residual ethanol. Cells were blocked with blocking buffer (3% BSA + 0.2% Triton X‐100 in PBS) at room temperature for 1 h, followed by incubation with primary antibodies at 4 °C for 12 h. After incubation, cells were washed five times with washing buffer (0.2% BSA + 0.05% Triton X‐100 in PBS) and incubated with fluorescence‐conjugated secondary antibodies for 1 h at room temperature in the dark. Following three additional washes with washing buffer under light‐protected conditions, nuclei were counterstained with DAPI (Southern Biotech 0100–20) before mounting for imaging using a confocal laser scanning microscope (ZEISS).

### Comet Assay

In A549 cells with endogenous LDHA knockdown and stable expression of either LDHA‐WT (wild‐type LDHA) or the LDHA‐2KR mutant, cells were treated with 30 µm cisplatin for 24 h and then collected as indicated. Subsequently, the cells were analyzed using a neutral comet assay kit. The detailed procedure was as follows: cells were mixed with low‐melting‐point agarose and applied onto comet assay‐specific slides (CometSlide), followed by incubation in lysis buffer at 4 °C for 1 h. The slides were then immersed in electrophoresis buffer for 45 min to allow DNA unwinding, and electrophoresis was performed at 20 V for 30 min. After electrophoresis, the slides were washed, dried, and stained with propidium iodide (PI) for 20 min. Excess stain was removed by rinsing with water, and the slides were visualized and imaged under a fluorescence microscope. Finally, comet tail moments were analyzed using OpenComet software.

### NHEJ Reporter Assays

A549 EJ5‐GFP reporter cells were seeded in 6‐well plates at a density of 20 000 cells per well. After pretreatment with sodium lactate (NaLa) or oxamate, 3 µg of I‐SceI plasmid was transfected into A549 EJ5‐GFP reporter cells using the Lipofectamine 3000 transfection kit (Invitrogen). Cells were harvested 48 h post‐transfection and analyzed by flow cytometry.

### Statistics and Reproducibility

All experimental procedures were performed in triplicate with consistent outcomes. Quantitative data represent mean values ± standard deviation (SD) derived from three or more independent replicates. Intergroup comparisons were evaluated using two‐tailed unpaired Student's *t*‐tests. Sample sizes were determined to ensure detection of effect sizes ≥1.5 with 80% statistical power and α = 0.05 significance threshold. Results with *p* < 0.05 were deemed statistically significant.

### Patient Samples

With proper ethical clearance (First Affiliated Hospital of Nanchang University Ethics Committee, CDYFYYLK (1‐19), 2020), paired LUAD and normal adjacent tissue samples were collected from consented patients at the First Affiliated Hospital of Nanchang University. All specimens were cryopreserved at −80 °C following collection for subsequent experimental use.

### Determination of IC_50_

A549 and H1299 cells were counted, 8000 cells/pores were inoculated into the 96‐well plate, the concentration gradient added cisplatin and Oxamate for three repeat pores respectively. After 24 h, MTT was added at the concentration of 10%, the culture medium was dried after incubation for 4 h, and 100 µL DMSO was added to dissolve the crystallization, then the absorbance was measured at the wavelength of 495 nm. The drug IC50 was analyzed by software.

### Mass Spectrometry‐Based Identification of Lactylated Proteins

To identify which proteins were lactated, A549 cells were lysed and the lysate products were purified using lactylation antibodies and agarose beads. Then the protein was separated on SDS‐PAGE and stained with Coomassie bright blue. The whole strip was cut off and sent to Jingjie Biological Company for mass spectrometry.

### Targeted Energy Metabolomics

Untargeted metabolomics was performed and analyzed by Metware.

## Conflict of Interest

The authors declare no conflict of interest.

## Author Contributions

J.L. and W.X. contributed equally to this work. J.W. and T.H. designed the study, J.L. and W.X. put the design into practice. J.L., W.X., T.Z., X.W., and R.L. designed and completed the experiments. Q.H., Y.W., and X.W. collected clinical tissues. J.L. and W.X. analyzed the data and wrote the manuscript. Z.Z. and J.Y. provided some of the plasmids required for the experiments. J.W. and T.H. revised the paper. This manuscript was approved by all authors.

## Supporting information



Supporting Information

Supporting Information

## Data Availability

The data that support the findings of this study are available from the corresponding author upon reasonable request.
