# Supplementary material for: Lactylation Enhances the Activity of Lactate Dehydrogenase A and Promotes the Chemoresistance to Cisplatin Through Facilitating DNA Nonhomologous End Junction in Lung Adenocarcinoma
Source: Adv Sci (Weinh). 2025 Nov 5;13(3):e10733. doi: 10.1002/advs.202510733 (PMC12806394; doi:10.1002/advs.202510733)
Supplement: Supplementary file 1 — Supporting Information [file ADVS-13-e10733-s002.docx]

**Supplementary information**


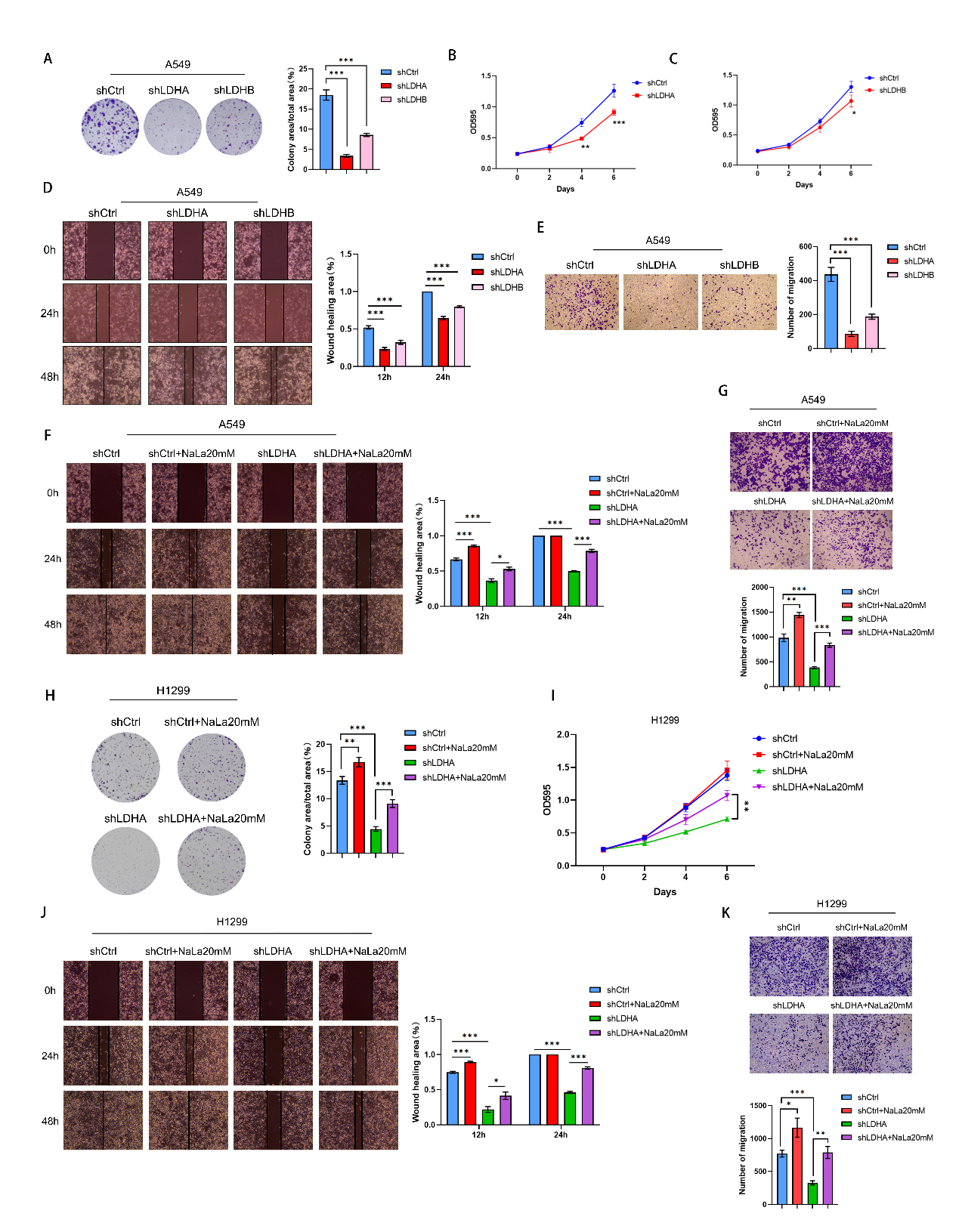
Fig. S1 LDHA, rather than LDHB, is the key factor driving global lactylation in LUAD cells. A. Colony formation of A549 cells after LDHA or LDHB knockdown. B.-C. Cell proliferation analysis of A549 cells following LDHA or LDHB knockdown. D. Wound healing capacity of A549 cells following LDHA or LDHB knockdown. E. Vertical migration capacity of A549 cells after LDHA or LDHB knockdown. F. Wound healing and Transwell migration assays (G) of A549-shCtrl and A549-shLDHA cells treated with or without 20 mM NaLa. H. Colony formation assay of H1299-shCtrl and H1299-shLDHA cells treated with 0 mM or 20 mM NaLa. I. Growth curve analysis of H1299-shCtrl and H1299-shLDHA cells treated with 0 mM or 20 mM NaLa. J. Wound healing and Transwell migration assays (K) of H1299-shCtrl and H1299-shLDHA cells treated with or without 20 mM NaLa. Data are presented as mean ± SD from three independent experiments. *p < 0.05, **p < 0.01, ***p < 0.001; ns stands for no significant change.


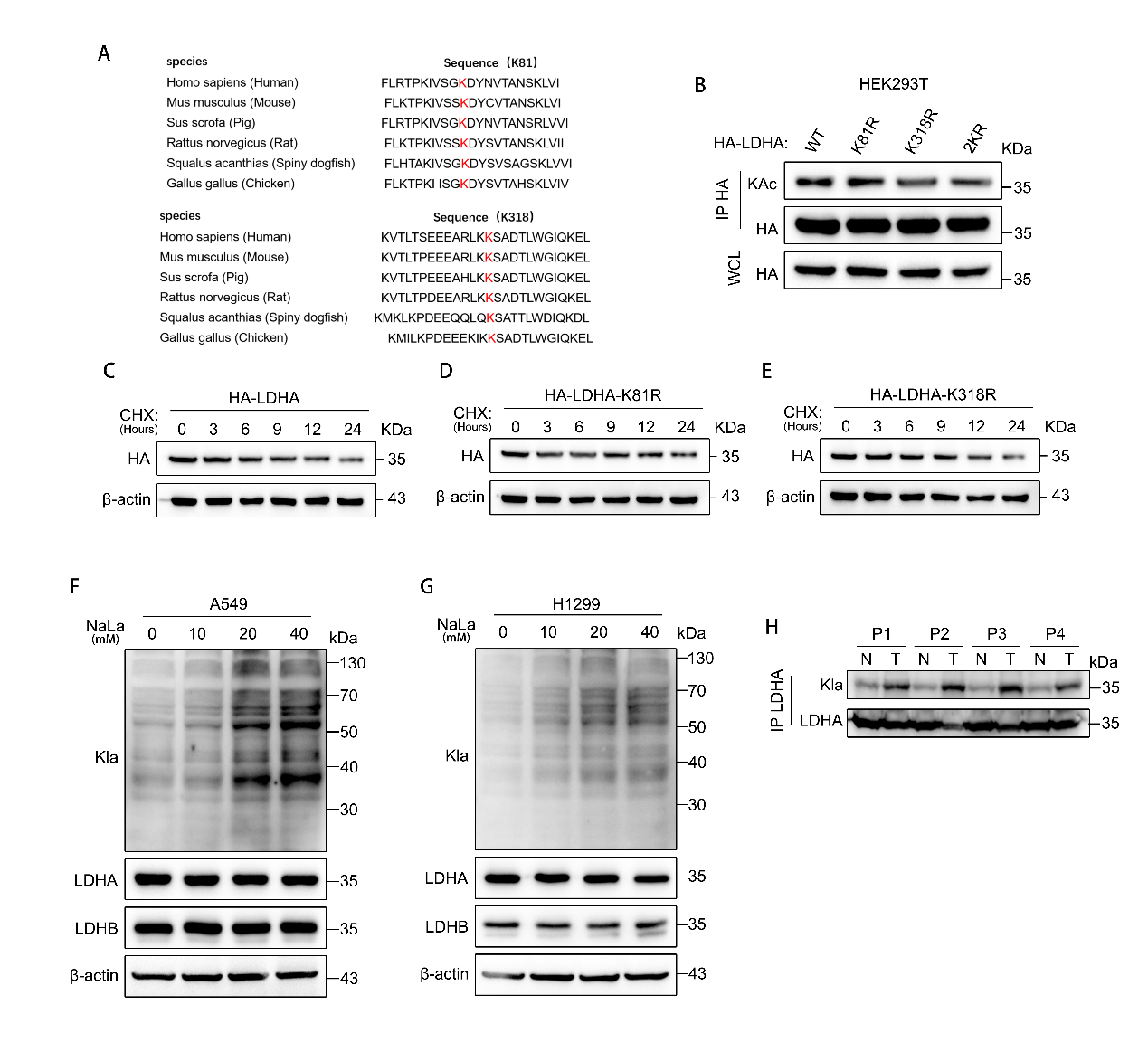
Fig. S2 Analysis of the Impact of Lactylation on LDHA Protein Function. A. Conservation analysis of K81 and K318 in LDHA across the indicated species. B. Acetylation analysis of LDHA-WT, LDHA-K81R, LDHA-K318R, and LDHA-2KR in HEK293T cells. C-E. A549 cells were transfected with wild-type HA-LDHA (LDHA-WT) or lactylation-deficient mutants (K81R and K318R), treated with cycloheximide (CHX) for indicated durations, and analyzed for protein degradation by Western blotting. F-G. Effects of NaLa treatment on LDHA, LDHB expression, and global protein lactylation in A549 and H1299 cells. H. The lactylation of LDHA was detected by immunoprecipitation in both tumor and adjacent normal tissues from patients 1 to 4.


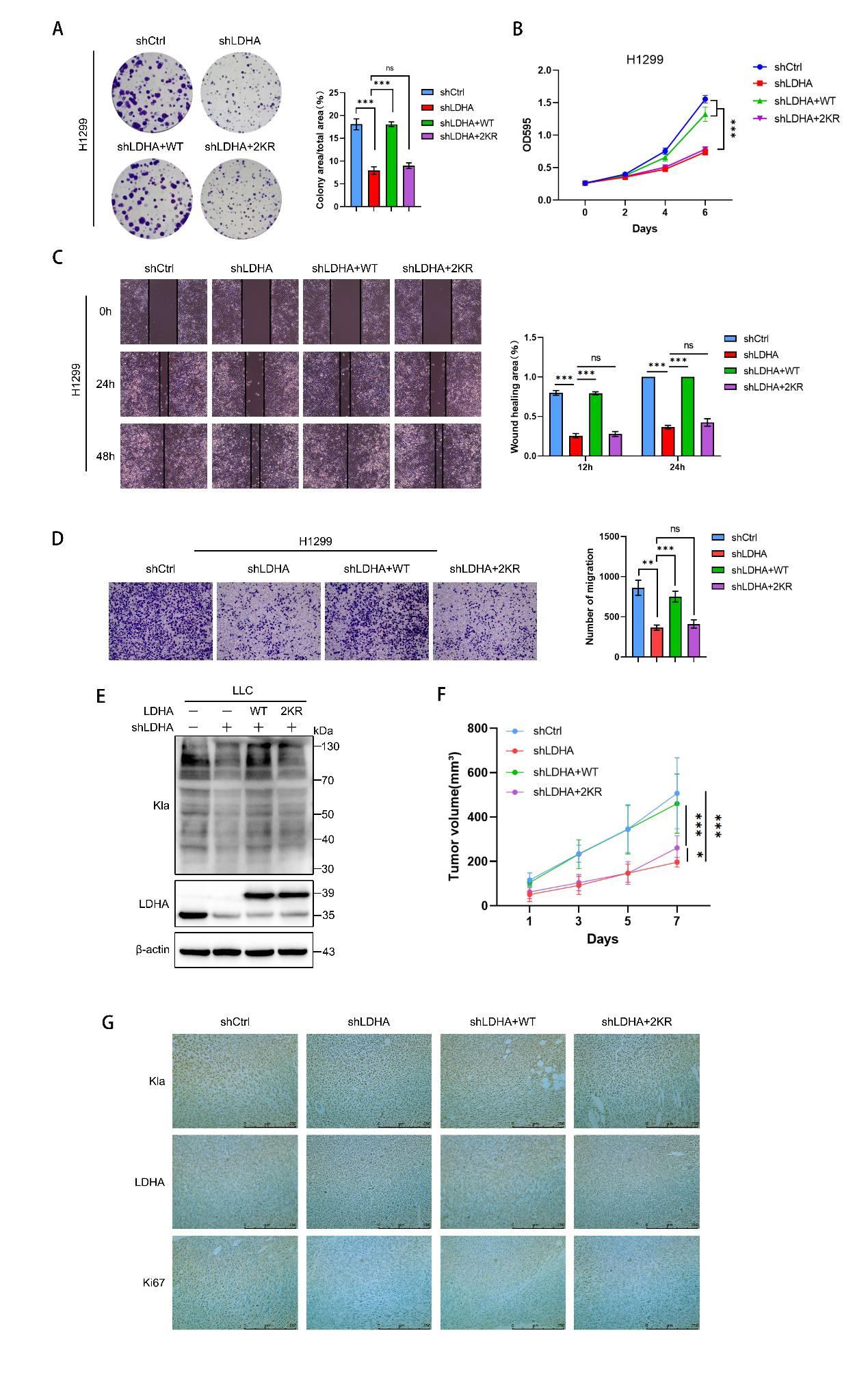
Fig. S3 The loss of lactylation in LDHA impairs its carcinogenic ability. A. Colony formation and growth assay (B) of H1299-shCtrl, H1299-shLDHA, H1299-shLDHA+WT, and H1299-shLDHA+2KR cells. C. Wound healing and Transwell migration assays (D) of H1299-shCtrl, H1299-shLDHA, H1299-shLDHA+WT, and H1299-shLDHA+2KR cells. E. Lactylation levels in LLC-shCtrl, LLC-shLDHA, LLC-shLDHA+WT, and LLC-shLDHA+2KR cells. F. Subcutaneous tumor growth curves of LLC-shCtrl, LLC-shLDHA, LLC-shLDHA+WT, and LLC-shLDHA+2KR cells in C57BL/6 mice. G. Immunohistochemical (IHC) staining of subcutaneous tumors in mice. Data are presented as mean ± SD from three independent experiments. *p < 0.05, **p < 0.01, ***p < 0.001; ns stands for no significant change.


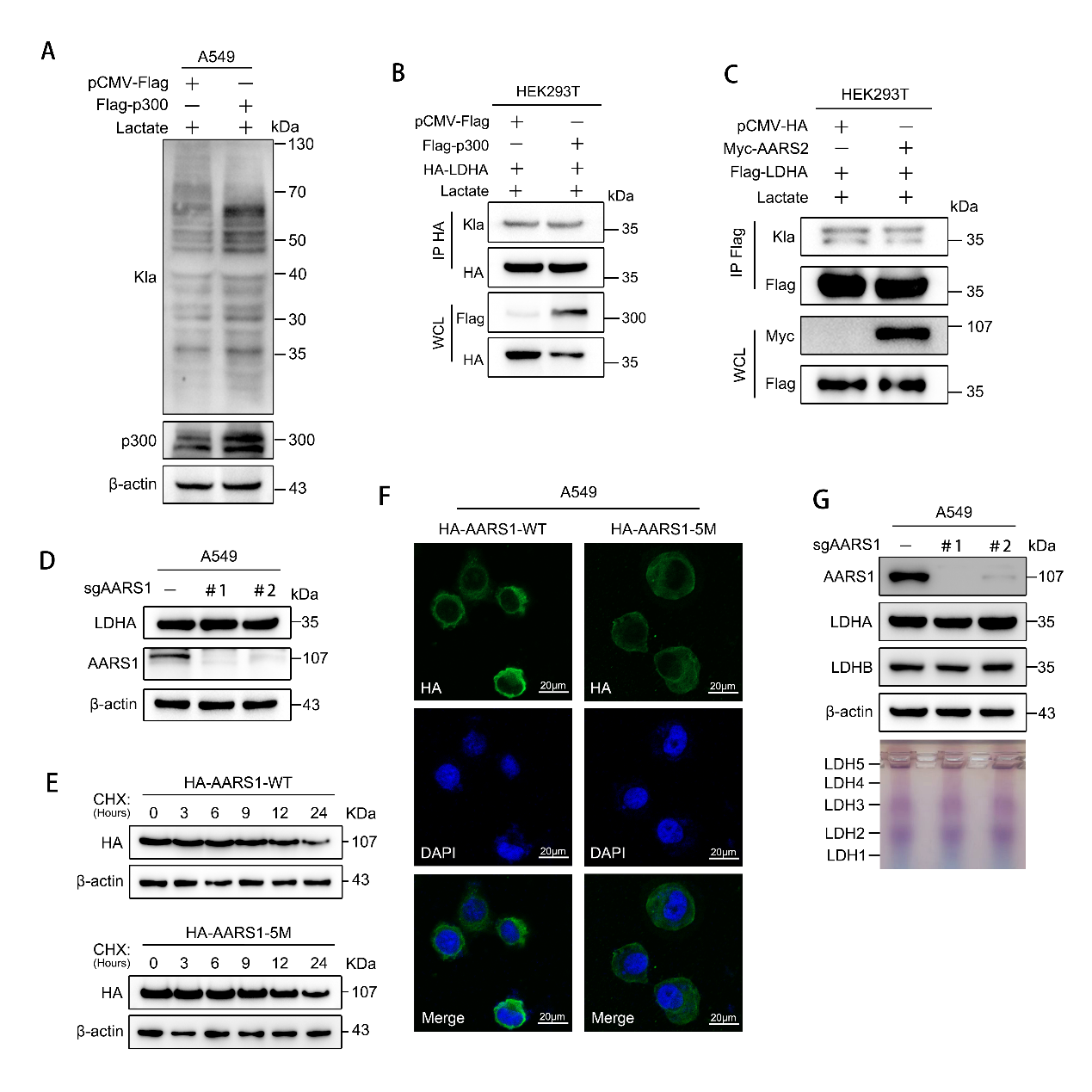
Fig. S4 Lactylation of LDHA mediated by AARS1 does not affect its protein expression, subcellular localization, or tetramer formation. A. Global lactylation profiling in p300-overexpressing A549 cells. B. Co-IP analysis of LDHA lactylation in HEK293T cells overexpressing p300 under lactate treatment. C. Co-IP analysis of LDHA lactylation in HEK293T cells overexpressing AARS2 under lactate treatment. D. Detection of LDHA protein expression in A549 cells with stable AARS1 knockout. E. The degradation rates of transfected AARS1-WT and AARS1-5M in A549T cells. F. Subcellular localization of transfected AARS1-WT and AARS1-5M was examined in A549 cells. G. In A549 cells with AARS1 knockout, protein expression was analyzed by immunoblotting. Following native gel electrophoresis, the gel exhibited distinct bands corresponding to LDH isoforms (LDH1 to LDH5).


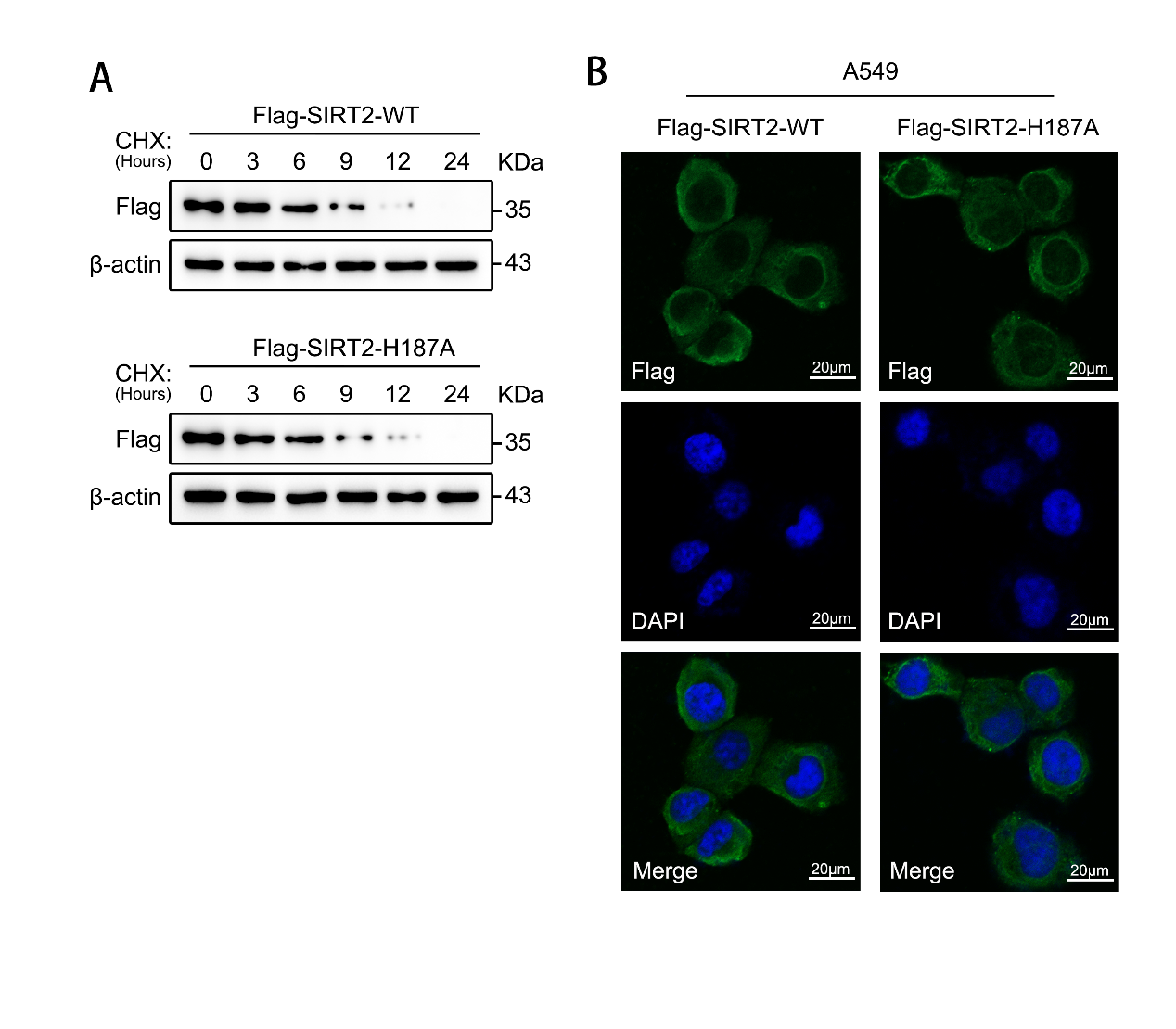
 Fig. S5 The H187A mutation in SIRT2 does not alter its degradation rate or subcellular localization. A. The degradation rates of transfected SIRT2-WT and SIRT2-H187A in A549T cells. B. Subcellular localization of transfected SIRT2-WT and SIRT2-H187A was examined in A549 cells.


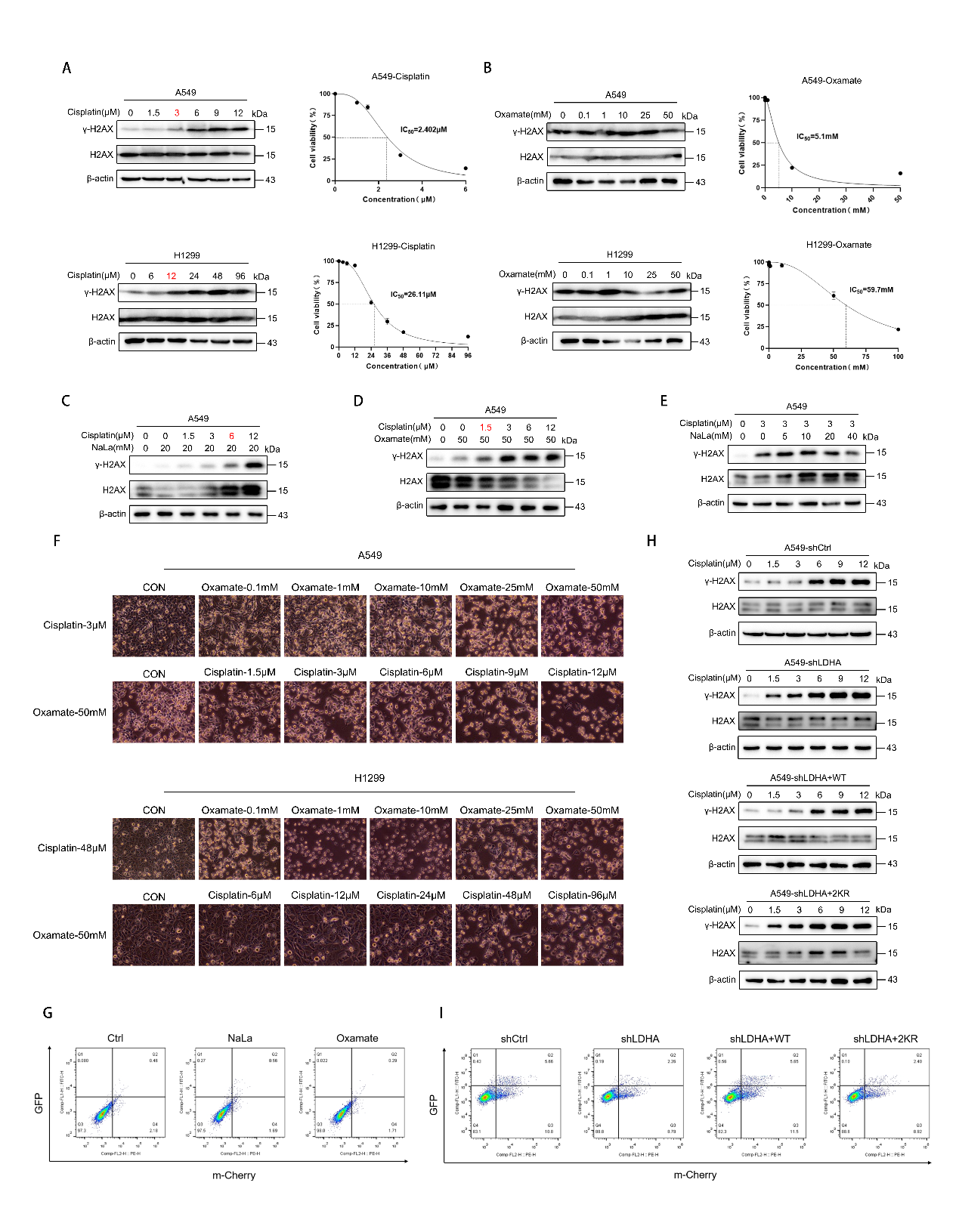
Fig. S6 LDHA affects the DNA damage response induced by cisplatin by regulating the overall lactate level of LUAD cells. A. DNA damage models were constructed using concentration gradient cisplatin in H1299 and A549 cells, while IC50 of cisplatin was measured in both cells. B. H1299 and A549 cells were treated with a gradient concentration of Oxamate and lysed. Western blot was performed to detect DNA damage, and the IC50 of Oxamate in both cells was measured. C. A549 cells were treated with 20 mM NaLa (24 h) and concentration gradient cisplatin (12 h), after which the cells were lysed, and protein immunoblotting was performed to detect DNA damage. D. The A549 cells were treated with 50 mM Oxamate (24 h) and concentration gradient cisplatin (12 h), and the cells were lysed, and protein immunoblotting was performed to detect the DNA damage. E. A549 cells were treated with concentration gradient NaLa (24 h) and 3 μM cisplatin (12 h), then lysed, and protein immunoblotting was performed to detect DNA damage. F. The A549 cells were treated with Oxamate (24 h) and 3 μM cisplatin (12 h), and 50 mM Oxamate (24 h) and cisplatin (12 h), respectively. H1299 cells were treated with concentration gradient Oxamate (24 h) and 48 μM cisplatin (12 h) and 50 mM Oxamate (24 h) and concentration gradient cisplatin (12 h), respectively, and white-light photographs were taken to observe the morphological changes and cell death. G. Flow cytometry analysis of Figure 6G. H. The LDHA-WT and LDHA-2KR plasmid were overexpressed in the stable cell line of shLDHA-A549, and the cells were treated with concentration gradient cisplatin (12 h), respectively, and protein immunoblots were performed to detect DNA damage. I. Flow cytometry analysis of Figure 6K.


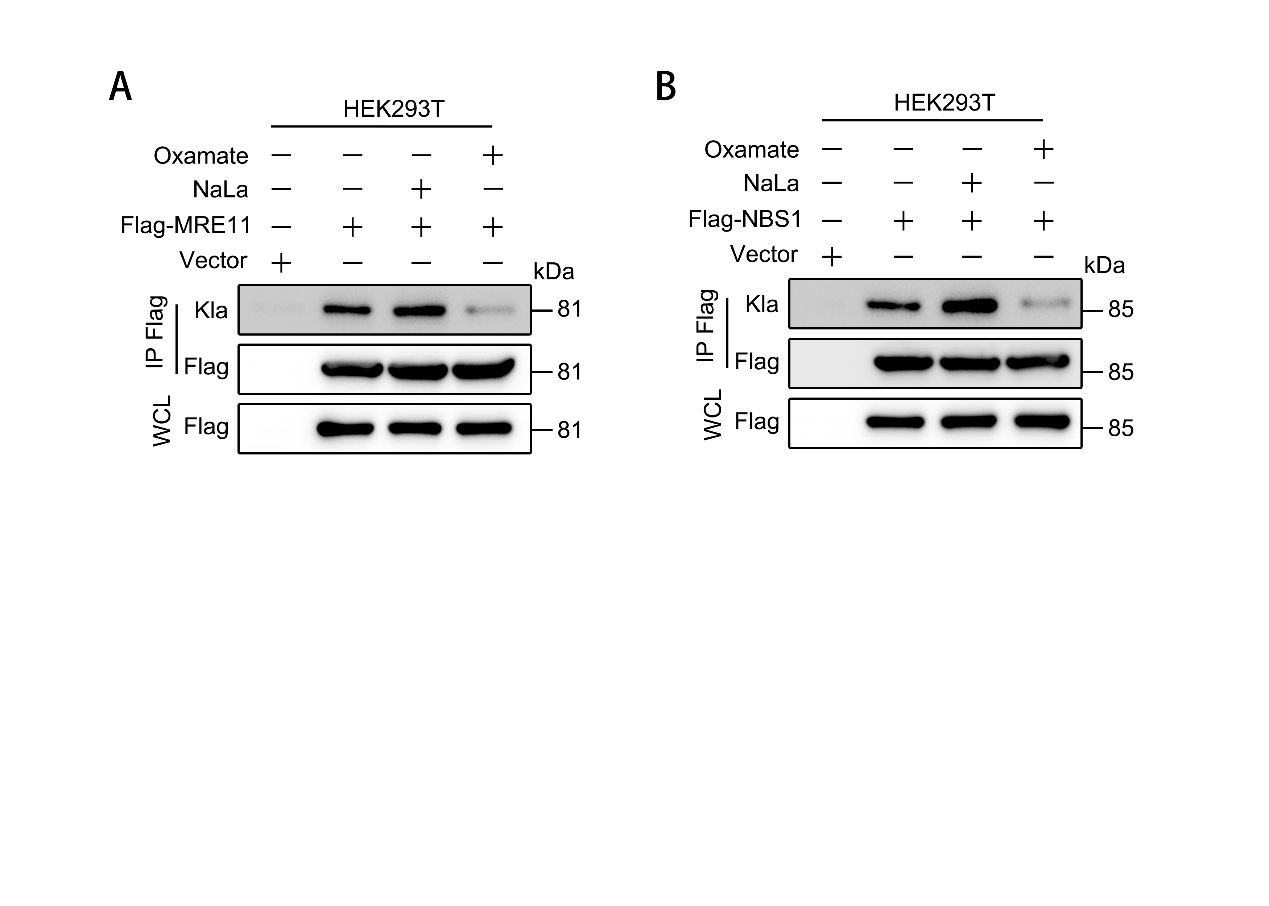
 Fig. S7 Detection of lactylation in other proteins based on mass spectrometry results. A. The lactylation of MRE11 was assessed in 293T cells following treatment with NaLa or Oxamate. B. The lactylation of NBS1 was assessed in 293T cells following treatment with NaLa or Oxamate.
