# Supplementary figures and images for: Lactylation Enhances the Activity of Lactate Dehydrogenase A and Promotes the Chemoresistance to Cisplatin Through Facilitating DNA Nonhomologous End Junction in Lung Adenocarcinoma
Source: Adv Sci (Weinh). 2025 Nov 5;13(3):e10733. doi: 10.1002/advs.202510733 (PMC12806394; doi:10.1002/advs.202510733)

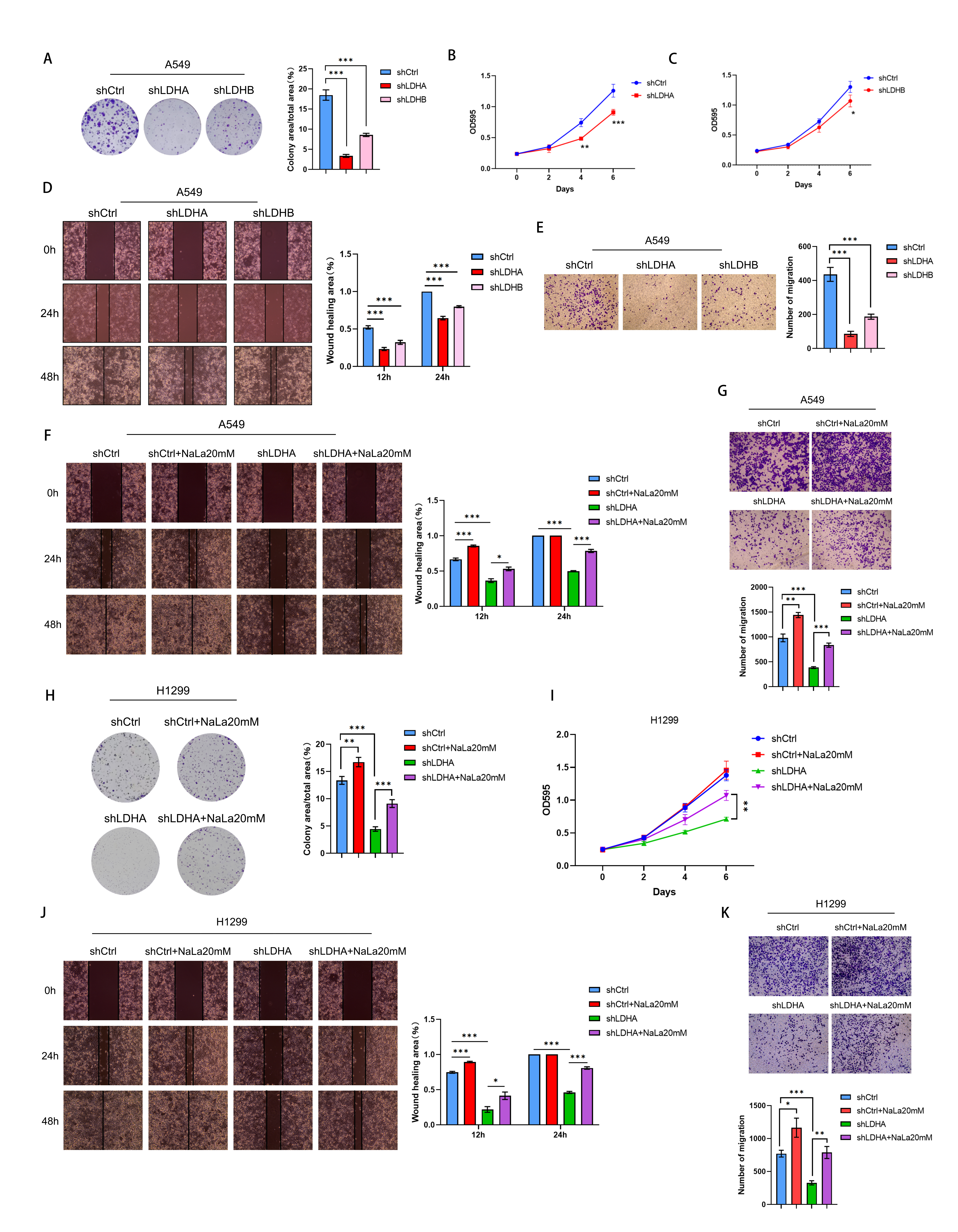

Supplement: Supplementary file 2 — Supporting Information [file ADVS-13-e10733-s001.zip › Fig S1-revised version.png]

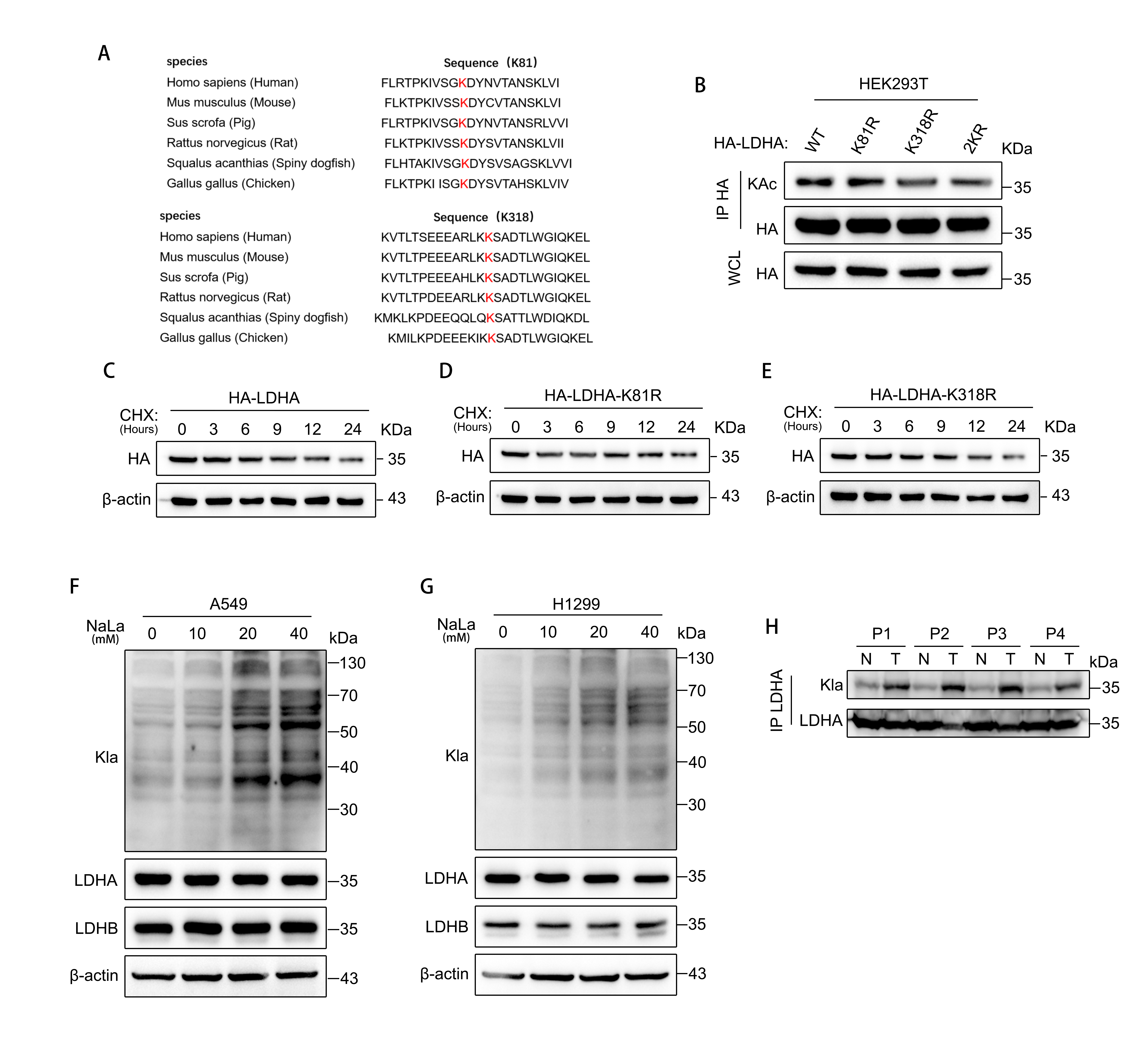

Supplement: Supplementary file 2 — Supporting Information [file ADVS-13-e10733-s001.zip › Fig S2-revised version.png]

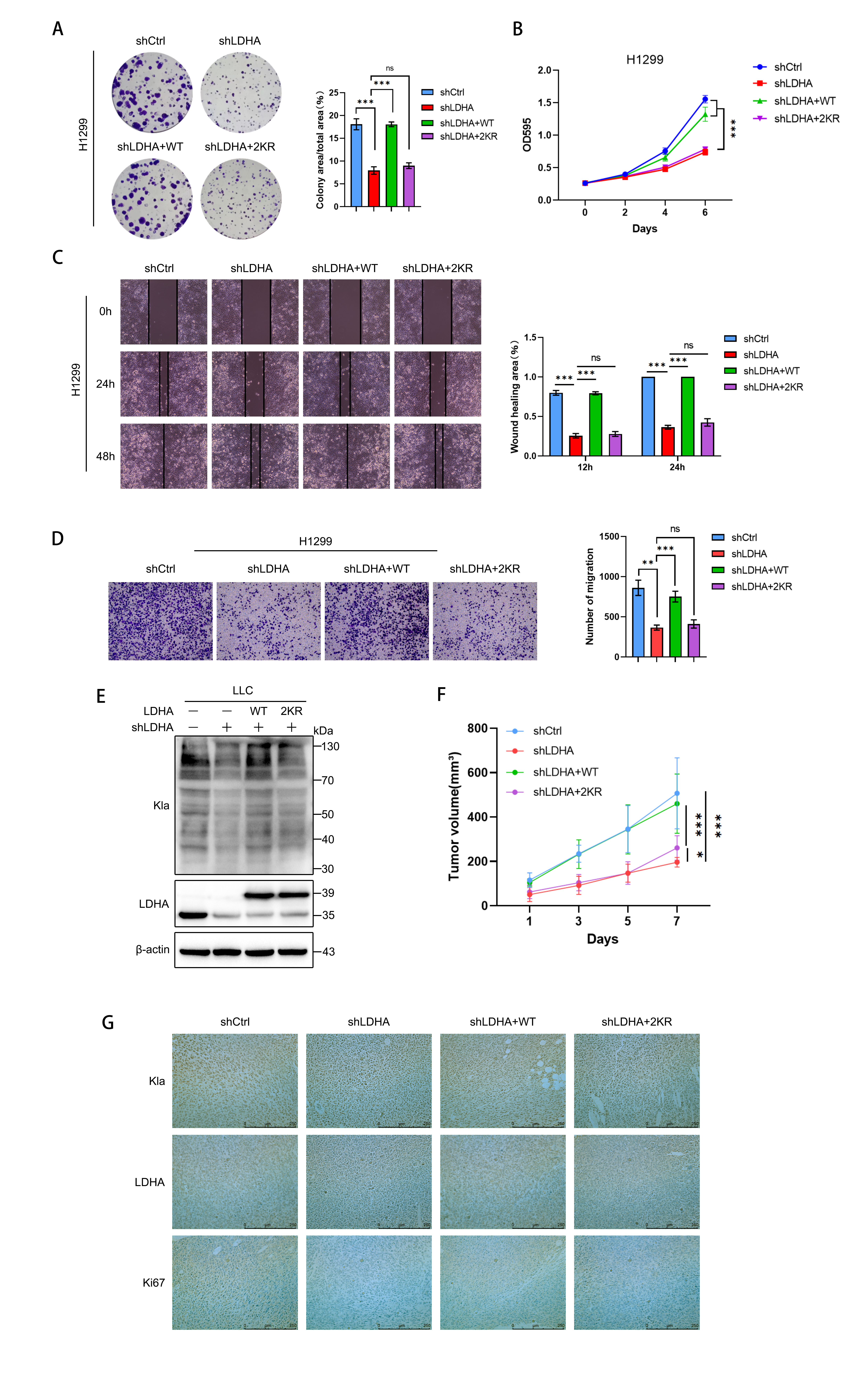

Supplement: Supplementary file 2 — Supporting Information [file ADVS-13-e10733-s001.zip › Fig S3-revised version.png]

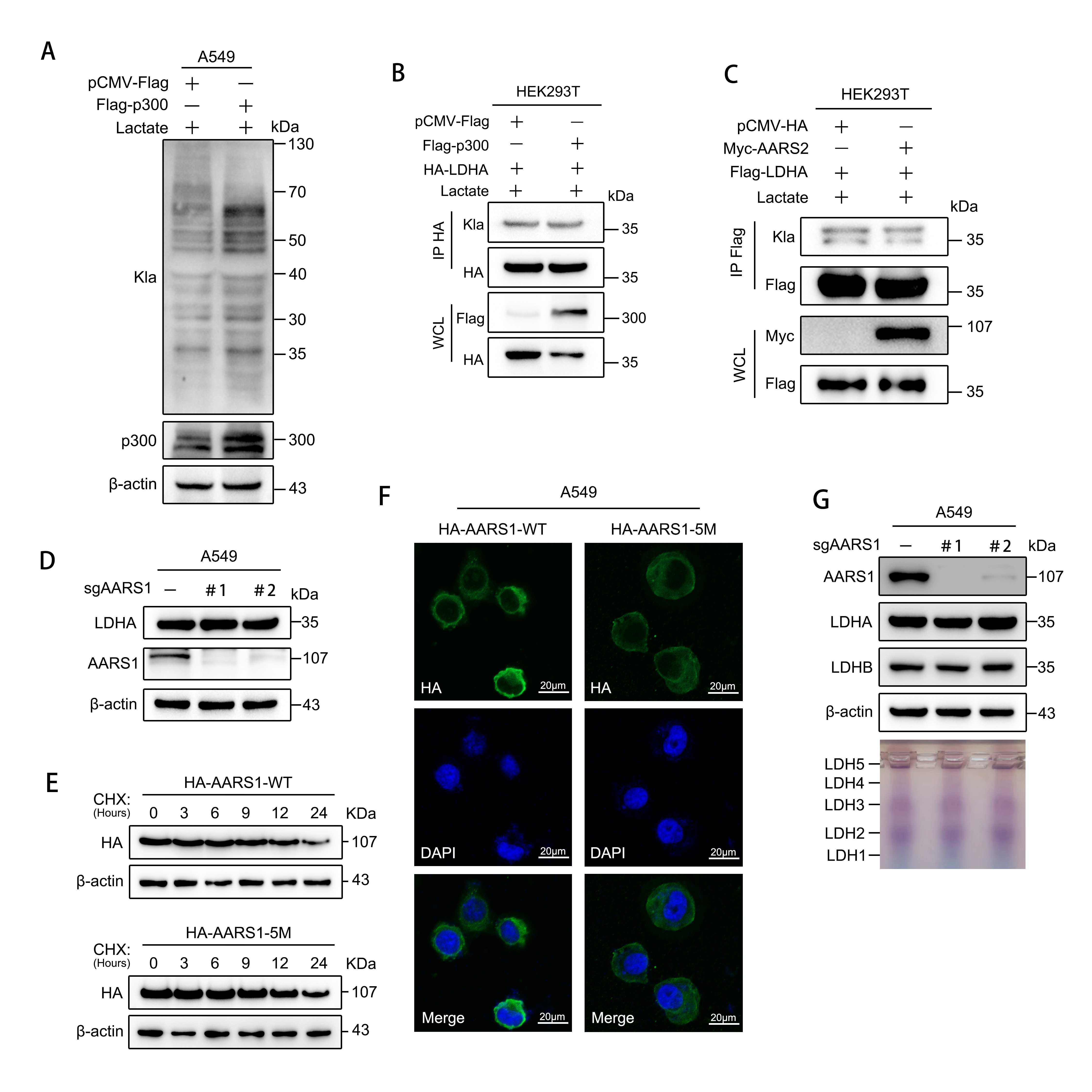

Supplement: Supplementary file 2 — Supporting Information [file ADVS-13-e10733-s001.zip › Fig S4-revised version.png]

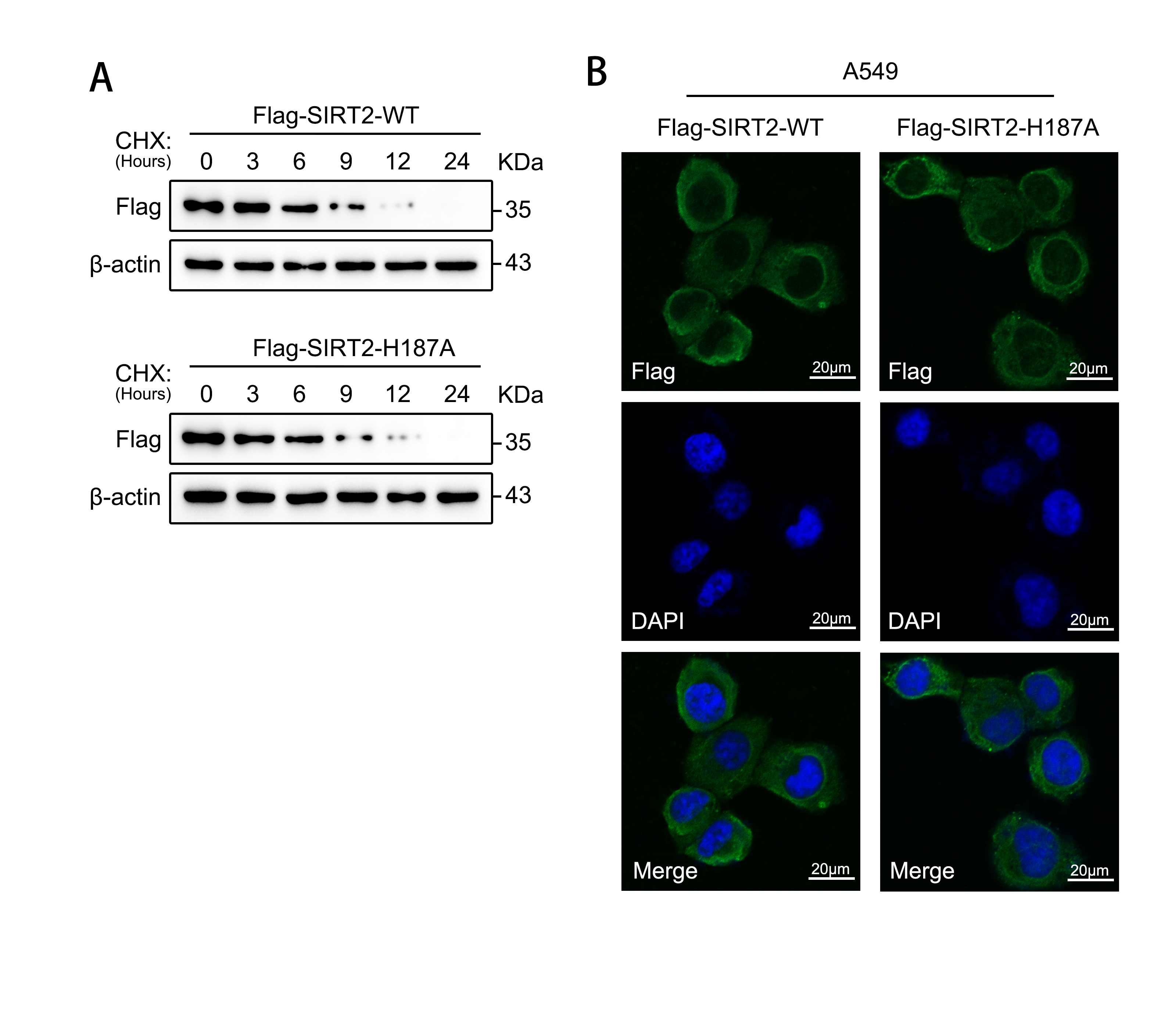

Supplement: Supplementary file 2 — Supporting Information [file ADVS-13-e10733-s001.zip › Fig S5-revised version.png]

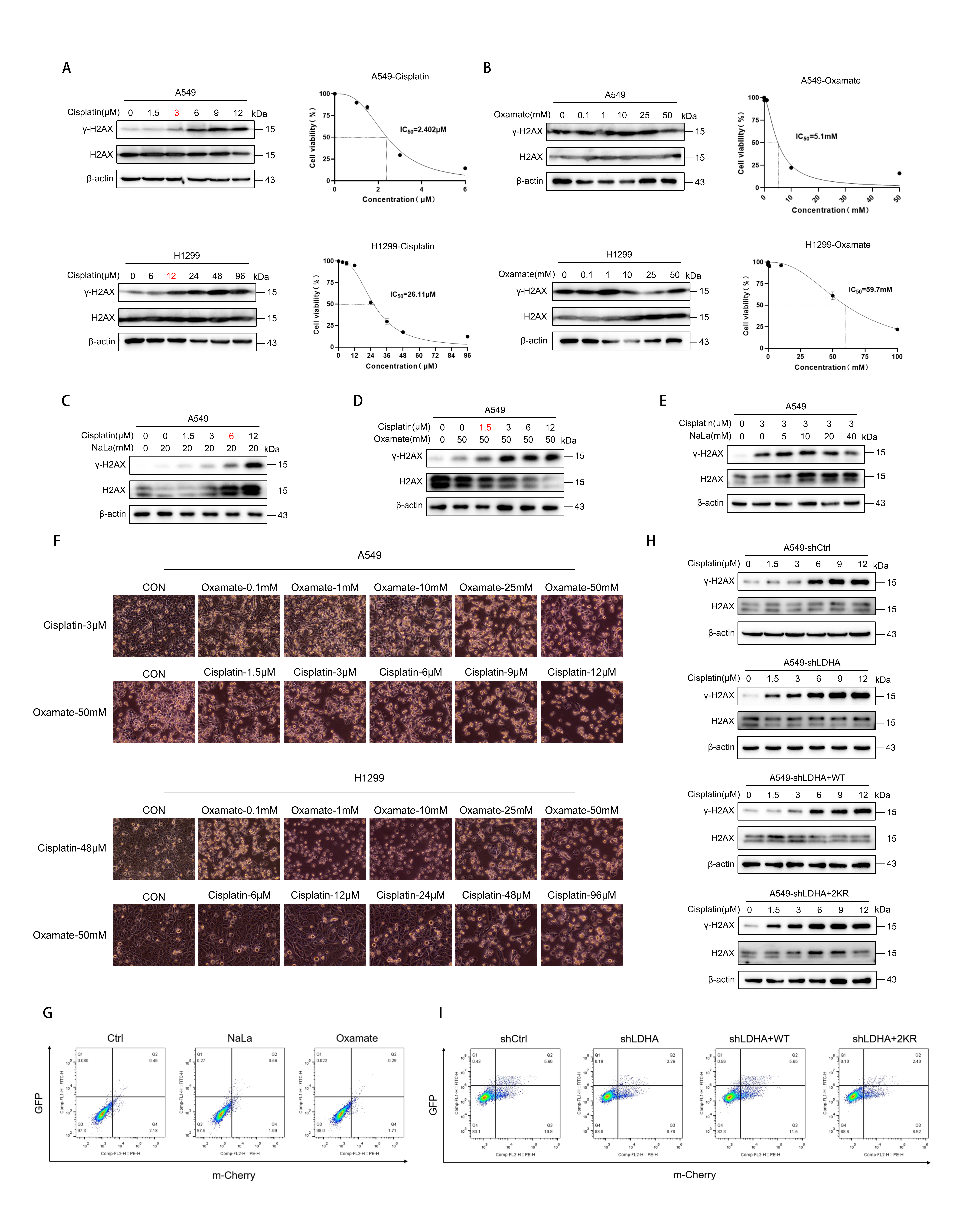

Supplement: Supplementary file 2 — Supporting Information [file ADVS-13-e10733-s001.zip › Fig S6-the latest revised version.png]

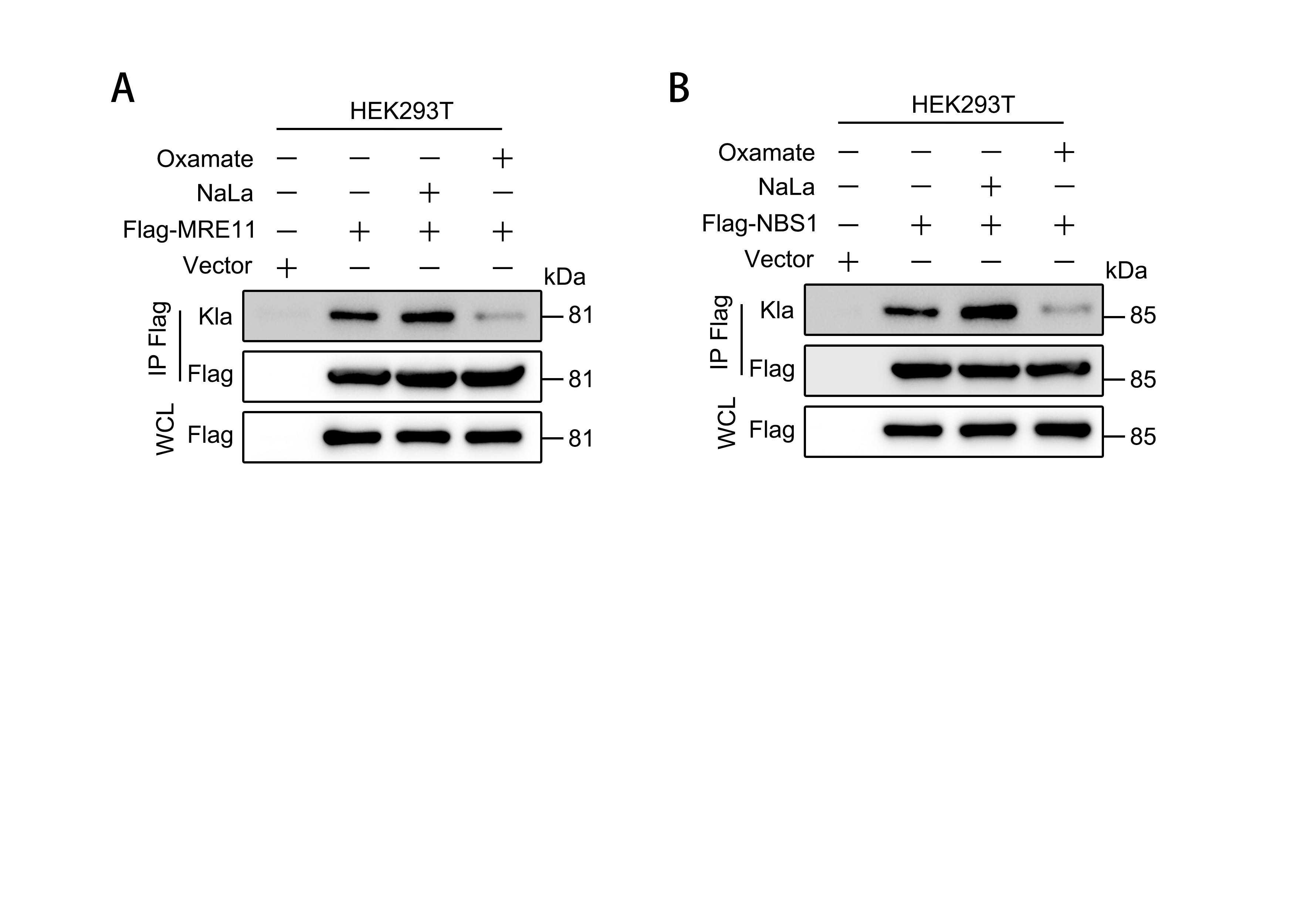

Supplement: Supplementary file 2 — Supporting Information [file ADVS-13-e10733-s001.zip › Fig S7-revised version.png]
